# Auditory Brainstem Responses in Tinnitus: A Review of Who, How, and What?

**DOI:** 10.3389/fnagi.2017.00237

**Published:** 2017-07-21

**Authors:** Victoria Milloy, Philippe Fournier, Daniel Benoit, Arnaud Noreña, Amineh Koravand

**Affiliations:** ^1^School of Rehabilitation Sciences, University of Ottawa Ottawa, ON, Canada; ^2^Centre National de la Recherche Scientifique, Aix-Marseille University Marseille, France

**Keywords:** tinnitus, ABR, review, brainstem, synaptopathy, meta-analysis, hearing loss

## Abstract

The auditory brainstem response (ABR) in tinnitus subjects has been extensively investigated over the last decade with the hopes of finding possible abnormalities related to the pathology. Despite this effort, the use of the ABR for tinnitus diagnosis or as an outcome measure is under debate. The present study reviewed published literature on ABR and tinnitus. The authors searched PubMed, MedLine, Embase, PsycINFO, and CINAHL, and identified additional records through manually searching reference lists and gray literature. There were 4,566 articles identified through database searching and 151 additional studies through the manual search (4,717 total): 2,128 articles were removed as duplicates, and 2,567 records did not meet eligibility criteria. From the final 22 articles that were included, ABR results from 1,240 tinnitus subjects and 664 control subjects were compiled and summarized with a focus on three main areas: the participant characteristics, the methodology used, and the outcome measures of amplitude and/or latency of waves I, III, and V. The results indicate a high level of heterogeneity between the studies for all the assessed areas. Amplitude and latency differences between tinnitus and controls were not consistent between studies. Nevertheless, the longer latency and reduced amplitude of wave I for the tinnitus group with normal hearing compared to matched controls was the most consistent finding across studies. These results support the need for greater stratification of the tinnitus population and the importance of a standardized ABR method to make comparisons between studies possible.

## Introduction

Tinnitus is known as a phantom sound that is perceived in the absence of an acoustic stimulation. It is described by patients in a variety of ways that can be as simple as a single pure tone and as complex as a combination of different sounds (Stouffer and Tyler, 1990). It can also be perceived differently in one ear, both ears or in the head, and can be modulated in some individuals by orofacial movements (Levine, 1999), touch, background noise, stress, anxiety, depression, and attention (Tyler et al., 2008).

Although, the pathophysiology of tinnitus is still not clear, various origins, and mechanisms have been described in the literature (Henry et al., 2014). The fact that tinnitus is not always suppressed when the cochlear nerve is sectioned suggests there are, at least, two distinct tinnitus sub-types: cochlear tinnitus and central tinnitus (House and Brackmann, 1981; Berliner et al., 1992). Cochlear tinnitus can be defined as a tinnitus subtype that results from aberrant activity in the cochlear nerve (Noreña, 2011). Central tinnitus can be defined as a tinnitus subtype that does not result from an increase of activity (or synchrony) in the cochlear nerve but rather at cortical levels within the central auditory pathways (Noreña, 2011). In this latter subtype, the tinnitus perception may result from cortical changes associated with the reduction of sensory inputs due to hearing loss.

One technique used to assess the activation along the neural pathways between the eighth peripheral nerve of the cochlear nucleus up to the inferior colliculus is the Auditory Brainstem Response (ABR) (Melcher and Kiang, 1996). Auditory Brainstem Responses (ABRs) are acoustically stimulated signals that represent the synchronized neural activation along the neural pathways. A study investigating the generation sites of ABRs in cats revealed the first wave (I) of the ABR reflects activity of the spiral ganglion cells at the distal part of the eighth auditory nerve, wave II is predominantly from the globular cells in the cochlear nucleus, wave III is generated by the cochlear nucleus spherical cells and globular cells, and waves IV and V generate from the medial superior olive and its projections to the nuclei in the lateral lemniscus and the inferior colliculus (Melcher and Kiang, 1996). These electrophysiological responses are typically less than a microvolt in amplitude (Burkard and Secor, 2002; Chalak et al., 2013). The success of revealing true and reliable responses relies heavily on averaging techniques employed to reduce noise contamination thereby improving the signal to noise ratio (Burkard and Secor, 2002). ABRs have been used clinically for two main purposes: hearing threshold estimations and neurodiagnostics. Indeed, the ABR is a well known cost-effective test that is routinely used in clinical practice as an objective diagnostic measure for determining the presence of hearing loss in infants, young children and patients that are difficult to test behaviorally. More so, the ABR is an important clinical tool for identifying the presence of retrocochlear lesions, acoustic neuromas, and vestibular schwannomas (Kotlarz et al., 1992; Rupa et al., 2003). This is achieved by identifying waves I, III, and V peaks and comparing the absolute latency values to normative ranges for each wave. For example, the presence of an acoustic neuroma at the level of the auditory nerve could significantly delay neural conduction. As a result, the latency between waves I and V is usually extended from the normative value by more than 0.2 ms (Wilson et al., 1992). Normative ABR latency values, for clicks at 70 dB nHL, collected on the most reliable waves I, III, and V are, respectively, 1.66, 3.68, and 5.64 ms for the left ear, and 1.66, 3.65, and 5.59 ms for the right ear (Chalak et al., 2013). When comparing between genders of the same age, the latencies are shorter and the amplitudes are larger in women compared to men (Hultcrantz et al., 2006). Hearing loss of different configurations affect the ABR: high frequency hearing losses show a delayed wave V at low intensities and a greater degree of wave I delay at all intensities, low frequency hearing losses show an earlier wave V at low intensities (Keith and Greville, 1987; Watson, 1996). Furthermore, elevated hearing thresholds also reduce the amplitude of waves I and V using the click-ABR (Sand and Saunte, 1994) and wave V using tone-burst ABR when the tone-burst characteristic frequency falls within the frequency region of the hearing loss (Lewis et al., 2015). The ABR sensitivity and specificity for both hearing threshold estimations and neurodiagnostics, have been shown to be very high with values of 100 and 91% for the former (Hyde et al., 1990) and 88 and 92% for the latter (Bauch et al., 1996). ABR assessment is also used for the diagnosis of auditory neuropathy (Starr et al., 1996). In such a case, the function of the outer hair cell of the cochlea is mostly normal, irrespective of hearing thresholds, even though the ABR waves are absent due to a lack of synchronized neural activity or excessive auditory fatigue (see Giraudet and Avan, 2012). The ABR technique thus provides information about the integrity of the central auditory system and can be a valuable diagnostic tool. Moreover, ABRs are relatively easy to obtain from only a few electrodes and are mostly insensitive to cognitive states (e.g., attention or arousal) or even consciousness (Burkard and Secor, 2002).

In tinnitus research, ABRs have been used in a variety of ways in humans. ABRs have been used to differentiate peripheral from central lesion sites in patients (Kehrle et al., 2008), and to investigate tinnitus treatment efficacy following drug administration (Shulman and Seitz, 1981; Milicic and Alcada, 1999; Bayar et al., 2001; Gopal et al., 2015). ABRs have also been used to identify noise-induced hidden hearing loss. In brief, Kujawa and Liberman found that the ABR wave I amplitude of mice significantly decreased at moderately-high levels (above 70 dB) up to 2 months following noise exposure even when the auditory thresholds had recovered to normal values (Kujawa and Liberman, 2006, 2009). In addition to the amplitude reduction, damage to the synaptic ribbons of the inner hair cells and spiral ganglion cells were revealed, suggesting that reduced wave I amplitude may be indicative of auditory nerve deafferentation. The term “cochlear synaptopathy” was further proposed to describe damage at the cochlear synapse without loss of hair cells resulting in “hidden hearing loss,” a functional hearing deficit without an elevation of audiometric thresholds (Liberman and Kujawa, 2017). In tinnitus patients with normal hearing (≤20 dB HL, Freq: 0.25–8 kHz), Schaette and McAlpine (2011) and Gu et al. (2012) showed similar reduced wave I amplitudes at high levels (80–90 dB SPL) compared to non-tinnitus matched controls, which were both interpreted as diminished activity of the low spontaneous rate auditory nerve fibers (LSR). Interestingly, the amplitude of wave V (measured baseline to peak) was reported to be significantly higher in only Gu et al. (2012). Schaette and McAlpine (2011) suggest the normal wave V amplitude, despite a reduction in wave I, is due to the central auditory system increasing its neural responsiveness to compensate for the reduced activity of the auditory nerve. Conversely, Gu et al. (2012) suggest that the higher amplitude of wave V is an artifact from the use of a lower frequency filter cutoff. Based on these findings, people suffering from tinnitus with normal audiometric thresholds show ABR amplitude changes that may be indicative of cochlear synaptopathy (reduced wave I) and the compensated responses of central/cortical regions (normal or elevated wave V). The increased responsiveness of central regions would generate increased spontaneous activity leading to tinnitus generation. Hickox and Liberman (2014) attempted to link synaptopathy to the generation of tinnitus in noise-exposed mice. The mice exposed to loud noise displayed the typical auditory nerve degeneration (determined by ribbon counts), reduced wave I amplitude/enhanced wave V ABR responses, and subtle changes in the behavioral response of tinnitus that did not reached significance (using the gap prepulse inhibition acoustic startle reflex or GPIAS). Low efficacy of this particular behavioral technique (GPIAS) in CBA-mice might explain the failure of significant results (Yu et al., 2016). Using another strain with better GPIAS could maximize these effects and link wave I reduction to a behavioral measure of tinnitus in animals. Another animal study (Rüttiger et al., 2013), exposed animals to loud noise and separated them based on tinnitus behavior. They found that although the ABR waveform was generally reduced after the trauma for both groups, wave I did not significantly change amplitude after recovery. Interestingly, the tinnitus group showed reduced wave IV and V amplitude after recovery, which the authors proposed to arise from a failure to compensate for the cochlear loss at the central levels of the auditory system.

It is noteworthy that ABR wave amplitude may be altered by the number of neural components activated by the stimulation and/or the level of synchronization between them. As amplitude of wave I is mostly due to tightly synchronized activity at the level of the cochlear nerve, the reduction in amplitude noted previously at high intensities might indicate not only a loss of neural fibers but also a decrease of synchronization. Conversely, increased neural synchrony has been proposed as a potential mechanism of tinnitus generation (Eggermont, 1984; Moeller, 1984). It was postulated that increased synchrony of the spontaneous firing rate even at the peripheral level of the auditory nervous system could be sufficient to produce a perception of a sound in the absence of external stimulation. The higher wave V amplitude reported in tinnitus subjects might reflect increased neural synchronization at higher levels of the auditory system. In brief, changes in wave I might reflect damage to the periphery and the following wave modifications might reflect compensation mechanisms such as higher increased neural synchrony in tinnitus. Still, modifications of wave III and wave V amplitude might occur without being related to wave I alterations. In a recent study, decreases in the amplitude of waves III and V were not adequately explained by changes in wave I in older participants compared to younger ones (Konrad-Martin et al., 2012). In this study, the reduction of the peak amplitudes of waves III and V, seemed to be linked to the effects of aging, instead of wave I amplitude reduction (and latency shift), which is believed to be the consequence of reduced auditory nerve inputs.

The current purpose of the study was to review ABR findings on tinnitus to assess any consistencies across studies in terms of absolute wave amplitudes and latencies. As ABR waves are affected by hearing loss (Don et al., 1998), and tinnitus mechanisms may differ between normal hearing and hearing loss participants (Henry et al., 2014), studies were separated based on this variable. A potential decrease in wave I amplitude in tinnitus subjects with normal hearing is expected to be one of the most consistent findings across studies (Schaette and McAlpine, 2011; Gu et al., 2012). The current review might also bring insight on possible modifications of the other waves such as wave III and wave V, in populations reporting tinnitus. A careful analysis of studies on tinnitus and ABR from 1980 to 2016 was made and convergent evidence was extracted based on the population/sample (who?), the methodology (how?), and the outcome (what?). The investigated outcomes were related to the latency and amplitude of waves I, III, and V.

## Methods

### Database search

**Figure 1:**
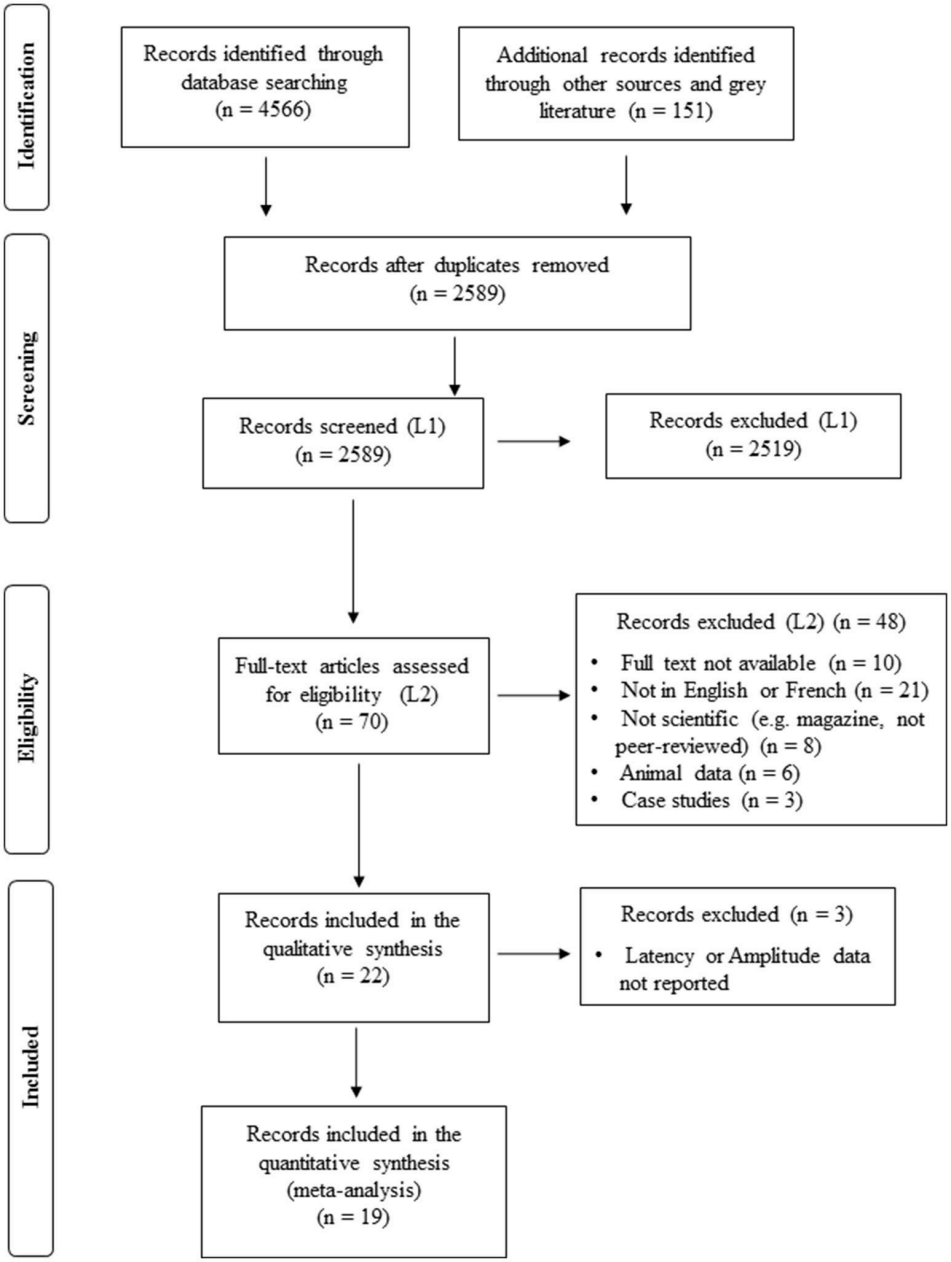
Flowchart of study selection (PRISMA diagram).

A scoping review of the literature was conducted using the method described by Arksey and O'Malley (2005). This approach uses a five stage framework that includes (1) identifying the research question, (2) identifying relevant studies, (3) selecting the study, (4) charting the data, and (5) collating, summarizing, and reporting the results. Statistics for each level of data collection are tabulated in the PRISMA schema (Moher et al., 2009; Figure 1).

A team approach to the process of the review was used to eliminate the level of error produced by a single individual and a second reviewer was used to independently analyze all the abstracts for inclusion (Levac et al., 2010). Given the high volume of articles yielded from the comprehensive search strategy, a “liberal accelerated” approach was used: the second reviewer analyzed only articles excluded by the first reviewer instead of the entire yield (Khangura et al., 2012).

The primary outcome of interest is measurement of the absolute peak amplitudes (peak to following trough) and latencies of the ABR waveform in tinnitus patients with and without hearing loss. Searches were conducted, between April 2015 and August 2016, by the principal investigator (V.M.) using the strategies detailed in Supplementary Table 1. In brief, the search terms (and their variations) used alone or in combination referring to tinnitus were: tinnitus, ear, buzz, ring, roar, click, pulsate, or pulse, and referring to the auditory brainstem measurements were: brainstem, brain, stem, auditory, response, potential, ABR, BAER, BSER, and evoked. The databases PubMed, CINAHL, Medline, PsycInfo, and Embase were searched separately and results were compiled in a Microsoft Excel (2011) spreadsheet where the search strategy yields were dated and organized (Supplementary Table 1). Gray literature which includes conference papers, master dissertations, and doctoral theses was also searched (October 2016) using ProQuest Dissertations and Theses Global and Conference Papers Index and added to the compiled list of articles. No conference papers, master dissertations, or doctoral theses were included in the final compilation as all those found were later published. Articles were limited to those published after 1980 as the waveforms were only just described in the 1970s by Jewett and colleagues and not yet applied to subjects with tinnitus (Jewett et al., 1970; Jewett and Williston, 1971). Any articles assessing populations with tinnitus due to underlying medical conditions were excluded for the purposes of this review (i.e., acoustic neuroma, otitis media, otitis externa, etc.). Excluding various comorbidities ensured the ABR outcomes reported were not due to known covariables. Any articles discussing ABRs for the purpose of measuring hearing loss such as threshold searching and newborn hearing screenings were also excluded. Preoperative and intraoperative ABRs were not included in this review as clinical populations with other underlying conditions, such as acoustic neuromas, or vascular abnormalities, are typically involved in these studies and are known covariables of ABR (Berliner et al., 1992; De Ridder et al., 2015).

The first screening (L1) consisted of excluding the articles that did not meet the criteria described in Table 1 based on the analysis of title and abstract of the article. The second reviewer screened the articles rejected by the first reviewer. Both reviewers completed the second screening (L2) where eligibility was based on the analysis of the full text. The use of a language translator was not feasible for this study, therefore all texts written in languages other than French or English were eliminated. Three case-report studies, where a single participant was reported (Shulman and Seitz, 1981; Milicic and Alcada, 1999; Gopal et al., 2015), were removed from the remaining yield. The sample size was too small to significantly contribute to the analysis, and the purpose of these studies was mostly to measure the responsiveness to treatment.

**Table 1:** Inclusion and exclusion criteria applied to the scoping review search.

	**Inclusion criteria**	**Exclusion criteria**
Population	Subjective tinnitus with or without hearing loss. This includes individuals with noise-induced hearing loss.	Subjective tinnitus with a history of ontological conditions (i.e., hypertension, tumors, demylenation, multiple sclerosis, Meniere's disease, auditory neuropathy, otitis media, otitis externa, middle ear pathologies etc.).
		Individuals with cochlear implants, CAPD, head trauma, psychological disorders, sudden hearing loss.
		Individuals with objective tinnitus or pulsatile tinnitus.
		Studies with a sample size of 1.
Evaluation	Auditory evoked potentials of the brainstem.	Long Latency Auditory Evoked Potentials (P1, N1, P2, N2); Event Related Potentials (MMN, P300, N400, P600 etc.). Auditory Steady State Responses, intraoperative ABR, preoperative monitoring, newborn hearing screening.
Publication type	Peer-reviewed journals only with articles published after 1980 in English or French.	Any unscientific papers: Magazine articles, conference proceedings, editorials, and manuals.
Outcomes	Measured peak amplitudes and latencies of the ABR wave.	

After the two levels of title and abstract screening (L1) and full-text screening (L2) were completed, a narrative synthesis was used to organize key points of the data into two charts. Key points of interest included population (i.e., age, tinnitus etiology, tinnitus localization, and hearing status), technical information (i.e., transducer used, ABR system used, stimulus type, presentation levels, tinnitus characterization, and recording filters), and results.

### Meta-analysis of the compiled data

The results were compiled in Microsoft Excel (2011) for a meta-analysis of the data using two different methodologies. The first method (meta-analysis 1) consisted of compiling the latency and amplitude values from Waves I, III, and V for all the subjects, with or without tinnitus, from all the studies reporting these values. The mean values for absolute latency and amplitude, standard deviations, and sample size for each study were organized in a table as a function of the ABR waves. The results were also separated based on hearing status and reported tinnitus. For example, the results of the four groups were determined based on the participants having (a) normal hearing without tinnitus, (b) normal hearing with tinnitus, (c) hearing loss without tinnitus, and (d) hearing loss with tinnitus. In these cases, we used the normal hearing and hearing loss definitions established within each article. These definitions varied from one study to the other (see Section Results and Discussion). Meta-analysis calculations were carried out on these data to determine (a) the total number of subjects for all the studies separated by hearing status; (b) the total mean latency/amplitudes weighted according to sample size of each study; (c) the composite standard deviation calculated as a combination of all the groups from all the studies; (d) the 95% confidence interval (CI) determined based on the composite standard deviation; (e) the mean difference latency/amplitude (i.e., the mean latency/amplitude of the tinnitus group subtracted by the mean latency/amplitude of the non-tinnitus group) again weighted according to the sample size. The confidence interval was calculated using Microsoft Excel software and consisted of adding or subtracting the confidence value from the weighted mean. The confidence value was calculated based on the composite standard deviation and the total number of observations of the pooled data grouped in one of the categories: normal hearing, normal hearing and tinnitus, hearing loss, or hearing loss and tinnitus. Confidence intervals were chosen because the interval estimate obtained from this method is more informative for data comparison of future studies than a sample mean and *T-*test. The confidence level of the confidence intervals was set at 95%, which is the equivalent of *p* < 0.05.

The second method (meta-analysis 2) consisted of calculating the difference of the mean amplitude and latency for waves I, III, and V (and the 95% CI) between the tinnitus and control groups, only for studies with at least matched age and hearing status. This method was added to minimize the risk of identifying differences in population variables, assessment techniques or methodologies between tinnitus and control groups. The differences found with the second methodology are thus presumed to be the result of group differences as both groups were tested with very similar protocols.

## Results

### Study selection

A total of 4,566 articles were retrieved from the databases PubMed, MedLine, Embase, PsycINFO, and CINAHL. An additional 133 articles were found by manually searching citations from the reference lists of articles that met the eligibility criteria and another 18 from gray literature (i.e., doctoral theses and conference papers). After the duplicates were removed, 2,589 articles were screened by title and abstract and 70 of those articles were analyzed by reading the full text. Of the remaining articles, 22 were included in the qualitative narrative synthesis and 19 in the meta-analysis (see Figure 1). The most common objective of the studies is the assessment of possible changes to the ABR of tinnitus patients compared to those without tinnitus (*n* = 19) for the purpose of distinguishing between peripheral and central tinnitus or identifying lesions or deafferentation of the auditory nerve. Other objectives were to compare the ABR to tinnitus perception (*n* = 1), emotions (*n* = 1), and the behavioral effects of residual inhibition (*n* = 1).

### Population characteristics

Characteristics of the populations tested in the 22 studies are shown in Table 2. Nineteen out of the 22 studies had a control group. The matching procedures varied between the studies, if mentioned at all. In 12 studies subjects were matched by sex, age, and hearing status, in three studies by age and hearing only and in four studies not matched (see Table 2). The mean age of the tinnitus and control groups was 40.1 and 38.0 years old, respectively, but varied widely between the studies ranging from 18 to 78-year-old participants. Since the data were not reported for smaller age groups, this data could not be separated into more narrowly defined age divisions. The tinnitus etiology was characterized as noise-induced for five of the studies, idiopathic for six studies, and not mentioned for the remaining 11 studies. Seventeen studies assessed patients with both bilateral or unilateral tinnitus, two studies used either bilateral tinnitus (Attias et al., 1993) or unilateral tinnitus exclusively (Maurizi et al., 1985) and three studies did not mention the lateralization of the tinnitus. Gender of the population was reported in 19 of the 22 studies of which five separated ABR data for males and/or females.

**Table 2:** Demographics include the number of subjects, the mean age, the tinnitus etiology, and localization and the hearing status criteria.

**Study**	**Subjects**	**Mean age in years (Range)**	**Tinnitus characterization**	**Tinnitus localization**	**Hearing status criteria**	**Results: latency**	**Results: amplitude**
**NOISE-INDUCED ETIOLOGY**							
Attias et al., 1993	Tinnitus (*n* = 12) Controls (*n* = 12) Matched: Age, HL severity and configuration	Not mentioned (26-45) Not mentioned (26-45)	Pitch Matching Loudness Matching	Bilateral (*n* = 12)	Audiometrically matched, No definition Noise induced hearing loss	No differences	No differences
Attias et al., 1996	Tinnitus (*n* = 13) Controls (*n* = 11) Matched: Age and Hearing	35 (21-45) Not mentioned (age and hearing matched)	Pitch Matching Loudness Matching Tinnitus severity profile	Unilateral (*n* = 8) Bilateral (*n* = 5)	Normal hearing: ≤20 dB HL, Freq: 0.25–2 kHz Hearing loss: 20–45 dB HL, Freq: 2–8 kHz	No differences	Enhanced Wave III amplitude
Gilles et al., 2016	Tinnitus (*n* = 19) Controls (*n* = 23) Matched: Age, Sex, and Hearing	~23 (SD: 2.4)	VAS–Loudness TQ	Head (*n* = 1) Unilateral (*n* = 2) Bilateral (*n* = 16)	Normal hearing (<25 dB HL, Freq: 0.25–16 kHz?) Hearing loss (>25 dB HL, Freq: 0.25–16 kHz?) HF tested (up to 16 kHz)	No differences	No differences
Santos-Filha et al., 2014	Tinnitus (*n* = 30) Controls (*n* = 30) Matched: Age, Sex, Hearing	41 (27-50) 41.6 (27-50)	VAS—Severity	Bilateral (67%) Unilateral (33%)	Normal hearing (<25 dB HL, Freq: 0.25–8 kHz)	No differences	Not reported
**IDIOPATHIC ETIOLOGY**							
Cartocci et al., 2012	Tinnitus (*n* = 10) Controls (*n* = 14) Matched: Age, Sex, Hearing	43.9 (SD: 11.0) 45.1 (SD: 11.9)	Not reported	Unilateral (*n* = 5) Bilateral (*n* = 5)	Normal hearing (≤20 dB HL, Freq: 0.125–8 kHz)	Longer Wave V and III–V	Not reported
Mahmoudian et al., 2013	Tinnitus (*n* = 44) No Controls	43.45 (18-65)	Not reported	Unilateral (*n* = 19) Bilateral (*n* = 25)	Included Hearing levels (≤30 dB HL, Freq: 0.5–2 kHz and <60 dB HL, Freq: 4–8 kHz)	No latency changes	III/V and I/V ratio modifications following electrical RI
Maurizi et al., 1985	Tinnitus (*n* = 54) No Controls	Not mentioned (23-78)	Residual Inhibition	Unilateral (*n* = 54)	Classification of hearing loss: 1) ≤20 dB HL, Freq: 0.5–4 kz 2) 21–49 dB HL, Freq: 0.5–4 kHz and >50 dB HL, Freq: 0.5–4 kHz	Prolonged Wave V in tinnitus ears vs. control ears	Not reported
McKee and Stephens, 1992	Tinnitus (*n* = 18) Controls (*n* = 19) Matched: Age and Hearing	26 (18-37) 27.5 (17-38)	Not reported	Head (*n* = 2) Unilateral (*n* = 2) Bilateral (*n* = 14)	Normal hearing (≤20 dB HL, Freq: 0.25–8 kHz) HF tested (up to 18 kHz)	No differences	Not reported
Nemati et al., 2014	Tinnitus (*n* = 25) Controls (*n* = 16) Matched: Age, Sex, Hearing	34.4 (20-57) Not mentioned (matched)	Not reported	Unilateral (*n* = 19) Bilateral (*n* = 6)	Normal hearing (<25 dB HL, Freq: 0.25–8 kHz)	No differences	Amplitude ratio V/I larger
Singh et al., 2011	Tinnitus (*n* = 25) Controls (*n* = 20) Matched: Age, Sex, Hearing	32 (18-45) Not mentioned (matched)	Not reported	Unilateral (*n* = 19) Bilateral (*n* = 6)	Normal hearing (<25 dB HL, Freq: 0.25–8 kHz)	Longer Wave I Shorter Wave V Shorter I–II and I–V	Not reported
**HETEROGENEOUS ETIOLOGY**							
Kim et al., 2016	Tinnitus (*n* = 123) No Controls	53.5 (SD: 13.4)	VAS—Discomfort Minimum masking level Residual inhibition THI Pitch matching	Unilateral (*n* = 79) Bilateral (*n* = 44)	Audiometric configurations: 1) Flat 2) High frequency gently sloping 3) High frequency steeply sloping	Prolonged latencies I, III and V for steeply high frequency hearing loss group	Not reported
**ETIOLOGY NOT MENTIONED**							
Barnea et al., 1990	Tinnitus (*n* = 12) Controls (*n* = ??) Matched: Age, Sex, Hearing	35 (21-45) Not mentioned (matched)	Pitch matching Loudness matching	Unilateral (60%) Bilateral (40%)	Normal hearing (≤20 dB HL, Freq: 0.25–8 kHz) HF tested (up to 20 kHz)	No difference	No differences
De Lavernhe-Lemaire and Beutter, 1989	Tinnitus (*n* = 164) Controls (*n* = 57) Not Matched	Not mentioned	Not reported	Unilateral (*n* = 112) Bilateral (*n* = 52)	Not mentioned	Longer Wave I, but decreased inter-peak I–V	Not reported
De Lavernhe-Lemaire and Beutter, 1990	Tinnitus (*n* = 139) Controls (*n* = 20) Not Matched	27-74	Not reported	Unilateral (*n* = 93) Bilateral (*n* = 46)	Not mentioned	Not reported	Decrease wave I and III amplitude
Gerken et al., 2001	Tinnitus (*n* = 9) Controls with normal hearing (*n* = 11) Controls with hearing loss (*n* = 8) Controls-elderly (*n* = 7) Not Matched	45.7 (26-68) 28 (22-37) 40.9 (23-53) 63.6 (60-68)	Pitch matching Loudness matching Minimum masking level	Not mentioned	Normal hearing (≤15 dB HL, Freq: 0.5–8 kHz) Hearing loss (>15 dB HL, Freq: 0.5–8 kHz)	Problem tinnitus group longer wave VII	No differences
Gu et al., 2012	Tinnitus (*n* = 15) Controls (*n* = 21) Matched: Age, Sex, Hearing	42 (SD: 6) 43 (SD: 7)	Pitch matching Loudness matching Minimum masking level Residual inhibition	Head (*n* = 4) Unilateral (*n* = 2) Bilateral (*n* = 9)	Normal hearing (≤20 dB HL, Freq: 0.25–8 kHz) HF tested (up to 16 kHz)	No latency differences	Reduced wave I and enhanced wave V
Ikner and Hassen, 1990	Tinnitus (*n* = 35) Controls (*n* = 35) Matched: Age, Sex, Hearing	40 36	Not reported	Not mentioned	Normal hearing (<20 dB HL, Freq: 1–4 kHz)	Longer wave I, III, V, and III–V interval	Not reported
Kehrle et al., 2008	Tinnitus (*n* = 37) Controls (*n* = 38) Matched: Age, Sex, Hearing	36 (SD: 7.2) Not mentioned (matched)	Not reported	Unilateral (*n* = 13) Bilateral (*n* = 24)	Normal hearing (<25 dB HL, Freq: 0.5–8 kHz)	Longer Wave I, III, V, and III–V	Ratio V/I
Kehrle et al., 2016	Tinnitus (*n* = 84) Controls (*n* = 47) Matched: Age, Sex, Hearing	37.2 (18-48) 35.7 (18-48)	VAS— severity Pitch matching Loudness matching THI	Unilateral (*n* = 26) Bilateral (*n* = 58)	Normal hearing (≤25 dB HL, Freq: 0.25–8 kHz)	Abnormal for wave I, wave III, wave V, inter-peak I–III, inter-peak III–V, Inter-peak I–V	Not reported
Lemaire and Beutter, 1995	Tinnitus (*n* = 355) Controls (*n* = 129) Not matched	52.1 (SD: 16.4) <25	Pitch matching Loudness matching	Unilateral (*n* = 220) Bilateral (*n* = 135)	Normal hearing (<20 dB HL, Freq: ??)	Longer for 0–I and I–V on the tinnitus affected side	Reduced Wave I, III, and sometimes V
Rosenhall and Axelsson, 1994	Tinnitus with hearing loss (*n* = 57) Tinnitus with normal hearing (*n* = 56) Controls with hearing loss (*n* = 166) Controls with normal hearing (*n* = 54) Matched: Age, Sex, Hearing	57.2 (SD: 10.6) 42.1 (SD: 13.8) Not mentioned (matched) Not mentioned (matched)	Not reported	Unilateral (*n* = 30) Bilateral (*n* = 83)	Normal hearing (<20 dB HL, Freq: 0.125–2 kHz and <35 dB HL, Freq: 4–8 kHz) Hearing loss (45-60 dB HL, Freq: 4 kHz)	Longer Wave I, III, and V	Not reported
Schaette and McAlpine, 2011	Tinnitus (*n* = 15) Controls (*n* = 18) Matched: Age, Sex, Hearing	36.3 (SD: 2.6) 33.2 (SD: 1.9)	Modified tinnitus spectrum	Not mentioned	Normal hearing (≤20 dB HL, Freq: 0.25–8 kHz) HF tested (up to 16 kHz)	Not reported	Reduced Wave I, No change Wave V

*SD, Standard deviation; dB, decibels; HL, hearing loss; Freq, frequency; n, number of subjects; VAS, visual analog scale; TQ, Tinnitus Questionnaire; THI, Tinnitus Handicap Inventory; HF, high frequency thresholds above 8 kHz*.

Hearing status was reported in all except two studies (De Lavernhe-Lemaire and Beutter, 1989, 1990). Among the studies that evaluated hearing, 17 studies used hearing status as a way to match controls to the tinnitus group. Three articles (Maurizi et al., 1985; Mahmoudian et al., 2013; Kim et al., 2016) did not have a control group and focused their comparisons on subgroups of tinnitus patients based on the configuration of the audiogram. These 17 studies either ensured that they used only a normal hearing population for both the tinnitus and control groups (*n* = 12), or a mixture of normal hearing and hearing loss for the control and tinnitus groups, matched based on the degree of hearing loss (*n* = 5). These studies used average audiometric thresholds of ≤15 dB HL (*n* = 1), <20 dB HL (*n* = 3), ≤20 dB HL (*n* = 6), <25 dB HL (*n* = 5), ≤25 dB HL (*n* = 1) as the criteria for normal hearing for the standard clinical frequencies. One study did not mention the criteria used to define normal hearing (Attias et al., 1993). Still, the frequency by which the audiometric criteria for normal hearing were applied varied from one study to the other. Indeed, there were typically defined from 0.25 to 8 kHz (*n* = 9), 0.125 to 8 kHz (*n* = 1), or 0.5 to 8 kHz (*n* = 2). Some studies limited the frequencies to a narrower range (*n* = 5). Of the five studies that used hearing loss populations, hearing loss was either undefined (Ikner and Hassen, 1990; Attias et al., 1993), defined within a limited range (i.e., 20-45, 21-49, 31-60, or 45-60 dB HL), or an unlimited range (i.e., above 15, 25 or 50 dB HL). A small number of studies (*n* = 5) tested frequencies above 8 kHz (Barnea et al., 1990; McKee and Stephens, 1992; Schaette and McAlpine, 2011; Gu et al., 2012; Gilles et al., 2016) (see also Table 2).

### Characteristics of the assessment and ABR technique

Tinnitus was characterized in only 12 of the studies (see Table 2). Of these studies, four used a visual analog scale to determine either loudness (*n* = 1), severity (*n* = 2), or discomfort (*n* = 1), and eight used matching psychoacoustic procedures for loudness and pitch or a variation called the modified tinnitus spectrum procedure where pitch and loudness are rated (see Table 2). Residual inhibition was measured in three studies. The characteristics of the assessment and the ABR technique can be found in Table 3. The most commonly reported systems used to acquire the ABR were the Nicolet CA-1000 (*n* = 4) and the Bio-Logic NavPro or Traveler Express (*n* = 5). Transducers used were typically the supra-auricular headphones TDH-39(P) (*n* = 6) and TDH-49 (*n* = 4), or the insert headphones ER-3(A) (*n* = 3) with the exception of one study that used high frequency Sennheiser HDA-200 circumauricular headphones (Gu et al., 2012). The stimulus type was largely broadband clicks (*n* = 19) with a typical duration of 0.1 ms (*n* = 16) presented at a rate of 10–31 clicks per second. Exceptionally, one study used 0.05 ms clicks (Schaette and McAlpine, 2011) and another study used 3 ms tone bursts (Gerken et al., 2001). Presentation levels were generally high (>80 dB) and were either expressed in HL (*n* = 8), nHL (*n* = 6), or SPL (*n* = 7). Of the six studies reporting stimulus level in dB nHL, three reported using their own subjects to determine the minimum click intensity in dB SPL that elicited a behavioral response (Maurizi et al., 1985; Gu et al., 2012; Mahmoudian et al., 2013). When filter characteristics were reported (*n* = 16), the cutoff frequency of the high-pass filters ranged from 5 to 200 Hz and from the 1,500 to 5,000 Hz for the low-pass filters. Contralateral masking was used in 5 studies, all of which used a white noise at an intensity of 55 dB nHL (Gilles et al., 2016) or 50 dB HL (De Lavernhe-Lemaire and Beutter, 1989, 1990; Lemaire and Beutter, 1995; Kehrle et al., 2008).

**Table 3:** Characteristics of the Auditory Brainstem Responses methodologies used including the latency and amplitude outcome results.

**Study**	**ABR system**	**Transducer**	**Polarity**	**Stimulus type**	**Presentation level(s)**	**Recording filters (Hz)**
Attias et al., 1993	Microshev-4000 System	Not reported	Alternating	Broadband clicks, (0.1 ms duration 10 clicks/s)	120 dB SPL	200–3,000
Attias et al., 1996	Not mentioned	TDH-49	Alternating	Broadband clicks, (0.1 ms duration 10.3 clicks/s)	120 dB peSPL	100–2,000
Barnea et al., 1990	Microshev-2000	TDH-49	Alternating	Broadband clicks, (0.1 ms duration, 10 clicks/s)	120 dB SPL	200–2,000
Cartocci et al., 2012	Epic Plus	Not reported	Alternating	Broadband clicks, (0.1 ms duration, 11 clicks/s)	90 dB HL 80 dB HL	150–1,500
De Lavernhe-Lemaire and Beutter, 1989	Nicolet CA-1000	TDH-39P	Alternating	Broadband clicks, (0.1 ms duration, 11.1 clicks/s)	90 dB HL	150–1,500
De Lavernhe-Lemaire and Beutter, 1990	Nicolet CA-1000	TDH-39P	Alternating	Broadband clicks, (0.1 ms duration, 11.1 clicks/s)	90 dB HL	150–1,500
Gerken et al., 2001	Tucker-davis FT5 Grass P511k	ER2 inserts	Not reported	Tone bursts (1–8 kHz) (3 ms duration, 9.7 clicks/s)	112.5 peak dB SPL	1–3,000
Gilles et al., 2016	Bio-Logic with Nav Pro	ER-3A	Alternating	Broadband clicks, (0.1 ms duration, 31 clicks/s)	80 dB nHL	100–3,000
Gu et al., 2012	Tucker-Davis Medusa	HDA-200	Condensation	Broadband clicks, (0.1 ms duration, 11 clicks/s)	30, 50, 70, 80 dB nHL	5–5,000
Ikner and Hassen, 1990	Nicolet CA-1000	TDH-39	Condensation	Broadband clicks, (0.1 ms duration, 21.9 clicks/s)	75 dB nHL	Not reported
Kehrle et al., 2008	Amplaid Mk-15	TDH-50P	Alternating	Broadband clicks, (0.1 ms duration, 12 clicks/s)	80 dB HL	100–2,500
Kehrle et al., 2016	Biologic Navigator Pro AEP	E-A-RTONE 3A	Rarefaction	Broadband clicks, (0.1 ms duration, 21.1 clicks/s)	80 dB HL	100–3,000
Kim et al., 2016	Not reported	Not reported	Not reported	Not reported	Not reported	Not reported
Lemaire and Beutter, 1995	Nicolet CA-1000	TDH-39P	Alternating	Broadband clicks, (0.1 ms duration, 11.1 clicks/s)	90 dB HL	150–1,500
Mahmoudian et al., 2013	Bio-Logic with Nav Pro	ER-3	Alternating	Broadband clicks, (0.1 ms duration, 11.1 clicks/s)	20 dB over the AEP threshold level	30–3,000
Maurizi et al., 1985	Amplaid MK 6	TDH-49	Rarefaction	Broadband clicks, (0.1 ms duration, 21 clicks/s)	60 dB nHL	200–2,000
McKee and Stephens, 1992	Biologic Evoked Potential System	Not reported	Not reported	Broadband clicks, (0.1 ms duration, 19.1 clicks/s)	85 dB HL	Not reported
Nemati et al., 2014	ICS CHARTR	Not reported	Alternating	Broadband clicks, (duration not reported, 11.1 clicks/s)	90 dB SPL	Not reported
Rosenhall and Axelsson, 1994	Madsen 2250 ERA	Not reported	Rarefaction	Broadband clicks, (duration not reported, 20 clicks/s)	80 dB nHL	150–2,000
Santos-Filha et al., 2014	Bio-Logic Traveler Express	TDH-39	Rarefaction	Broadband clicks (0.1 ms duration 19 clicks/s)	80 dB HL	Not mentioned
Schaette and McAlpine, 2011	Medelec Synergy T-EP system	TDH-49	Not reported	Broadband clicks, (0.05 ms duration, 11 clicks/s)	90 and 100 dB SPL	100–1,500
Singh et al., 2011	Not reported	Not reported	Not reported	Not reported	Not reported	Not reported

*dB, Decibels; HL, hearing loss; SPL, sound pressure level; TDH, Telephonics supraauricular headphones; ER, Etymonic insert earphones; HAD, Sennheiser circumauricular headphones*.

Latency was reported in all studies except for De Lavernhe-Lemaire and Beutter (1990) and Schaette and McAlpine (2011). The most common outcome was no change in latency (*n* = 9) or an increase in the latency of waves I (*n* = 8), III (*n* = 5), and V (*n* = 7) for the tinnitus group (See Table 2). Only one study reported a decrease in wave V latency. Other latency changes for the tinnitus group varied considerably from increased interlatencies between waves III–V (*n* = 4), I–V (*n* = 1), and I–III (*n* = 1) to decreased interlatencies between waves I–II (*n* = 1) and I–V (*n* = 3). Out of the 12 studies that reported amplitude, four did not report any changes. The others reported the tinnitus group amplitudes either increased for waves III (*n* = 1), or decreased for waves I (*n* = 4), III (*n* = 2) and V (*n* = 2). Amplitude ratios were reported in four studies: V/III (*n* = 1) and V/I (*n* = 3). Gilles et al. (2016) was the sole study that reported the latency and amplitude of waves II and IV.

### Meta-analysis 1: quantitative analysis of ABR latency and amplitude changes separated by hearing status

The data of the 19 studies were compiled: a total of 1,240 subjects included in the tinnitus population and 664 control subjects were found. Three studies were not included because they have not reported any amplitude or latency data in format suitable for the analysis (see Figure 1). A summary of the mean latency and amplitude pooled from all studies is presented in Table 4. Each ear was treated as a separate data point when available in the literature. The raw latency and amplitude values for all the studies are available in Supplementary Table 2. Table 4 shows that for the normal hearing populations, there is no significant difference between the tinnitus and non-tinnitus groups. The difference in mean latency for the normal hearing tinnitus group was 0, 0.01, and 0.03 ms higher than the control group for waves I, III, and V, respectively. For the hearing loss populations, the tinnitus group lower limit (95% CI) values of 1.75 (I), 3.83 (III), and 5.80 (V) ms were significantly higher than the upper limit (95% CI) values of 1.62 (I), 3.76 (III), and 5.68 (V) ms for the non-tinnitus groups. When comparing hearing loss groups, the tinnitus groups were 0.21, 0.15, and 0.22 ms delayed for waves I, III, and V compared to the group without tinnitus. Amplitudes for the normal hearing population were 0.04 μV lower for the wave I, and 0.02 and 0.01 μV higher for waves III, and V, respectively, for the tinnitus group. The hearing loss population showed 0.1, 0.09, and 0.06 μV lower wave I, III, and V amplitudes for the tinnitus group. Amplitudes were significantly different for the hearing loss population but not significantly different for normal hearing populations. Nevertheless, the wave I amplitude reduction in the tinnitus with normal hearing compared to the normal hearing controls was close to significance with a higher limit of the 95% CI of 0.24 μV compared to 0.23 μV 95% CI lower limit for the controls.

**Table 4:** Summary table of the meta-analysis (1) of the mean latency and amplitude of waves I, III, and V for tinnitus and non-tinnitus groups separated by hearing status.

	**Tinnitus**			**No tinnitus**		
	**I**	**III**	**V**	**I**	**III**	**V**
**NORMAL HEARING**						
Mean latency (ms)	1.59	3.73	5.61	1.59	3.72	5.58
Standard error	0.02	0.02	0.02	0.01	0.02	0.02
95% CI	1.55–1.62	3.69–3.76	5.56–5.65	1.58–1.61	3.68–3.75	5.53–5.62
*N*-value	142	142	152	490	118	132
Mean Amplitude (μV)	0.21	0.34	0.43	0.25	0.32	0.42
Standard error	0.01	0.01	0.02	0.008	0.009	0.01
95% CI	0.19–0.24	0.31–0.36	0.39–0.48	0.23–0.26	0.30–0.34	0.40–0.44
*N*-value	105	75	75	248	212	212
**HEARING LOSS**						
Mean latency (ms)	1.77	3.86	5.84	1.56	3.71	5.62
Standard error	0.007	0.02	0.02	0.03	0.03	0.03
95% CI	**1.75**–1.78	**3.83**–3.89	**5.80**–5.88	1.5–**1.62**	3.66–**3.76**	5.57–**5.68**
*N*-value	1,407	369	385	69	69	69
Mean Amplitude (μV)	0.15	0.19	0.33	0.25	0.28	0.39
Standard error	0.004	0.004	0.005	0.04	0.03	0.03
95% CI	0.14–**0.16**	0.18–**0.20**	0.32–**0.34**	**0.17**–0.32	**0.23**–0.34	**0.34**–0.45
*N*-value	831	919	919	34	34	34

*Confidence intervals (CI) and number of observations (n-value) are presented for each group. Bolded values are significant (comparing the limits of the 95% CI of the tinnitus to the no tinnitus group). Number of observations was obtained by adding the reported number of subjects or ears of each study within the group*.

### Meta-analysis 2: quantitative analysis of ABR latency and amplitude mean difference between tinnitus and matched control groups separated by hearing status

The mean differences in latencies and amplitudes between the tinnitus group and the matched controls (age and hearing status) were extracted when possible. For the latencies, the extraction of the mean difference was possible for only 10 out of the 15 studies that minimally matched their controls for age and hearing status. From the five excluded studies, one study did not report latencies (Schaette and McAlpine, 2011) and four studies provided insufficient information for data extraction (i.e., only the tinnitus data were presented, standard deviation was omitted, etc.; Barnea et al., 1990; McKee and Stephens, 1992; Rosenhall and Axelsson, 1994; Nemati et al., 2014). To note, only two studies out of the 10 studies that were kept for the latency meta-analysis did not include gender in their matching procedure (Attias et al., 1993, 1996). Interestingly, they were also the two studies with the highest degree of variability for both latency and amplitude analysis. For amplitudes, the number of included studies is even lower with five studies included in the second meta-analysis. Given that the amplitudes of waves III and V are poorly reported, the analysis was made on wave I only. Overall, similar problems extracting sufficient information were found for both the amplitude and latency data.

**Figure 2:**
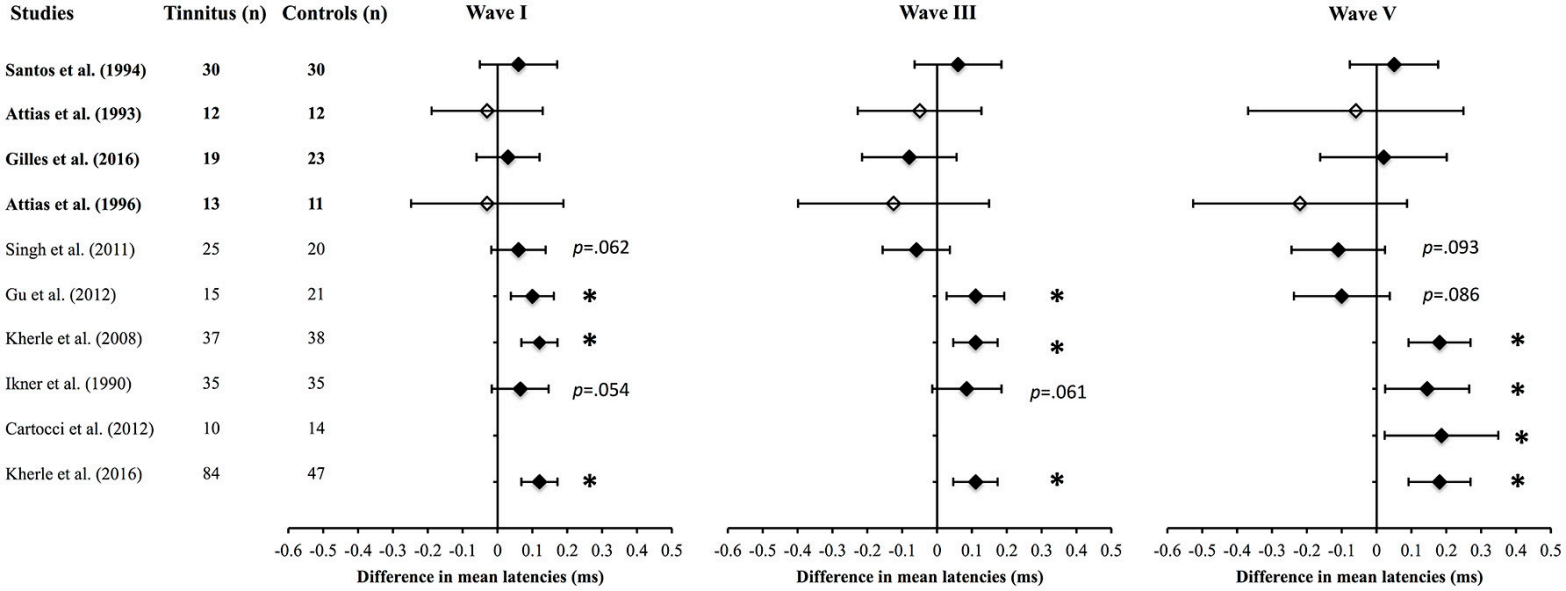
Meta-analysis (2) of the net difference in latencies for Wave I, III, and V (95% CI) between tinnitus and control groups when matched for hearing status. Black diamonds represent studies with normal hearing participants and white diamonds studies with hearing loss participants. The studies in bold included only noise-induced tinnitus. ^*^Asterisks represent significant differences (*p* < 0.05).

For the latency mean differences, the meta-analysis revealed that only three studies out of 10 found a significantly prolonged wave I in the tinnitus group compared to controls and two other studies were close to significance (Figure 2). To note, two of the studies that are not close to significance were the only ones that tested participants (tinnitus and controls) with hearing loss (Figure 2, white diamonds), all the other studies used normal hearing individuals for both the tinnitus and control groups (Figure 2, black diamonds). As previously mentioned, they were also the two only studies that did not match their groups on the basis of gender. For wave III and V latencies, significantly prolonged latencies were found in three studies for the former and four for the latter. Kehrle et al. (2008, 2016) were the only studies that showed all three waves were significantly prolonged although Ikner and Hassen (1990) reported a similar trend. Interestingly, none of the studies with a specific noise induced tinnitus etiology inclusion criteria reported a significant latency effect for any of the waves (Figure 2, studies in Bold).

**Figure 3:**
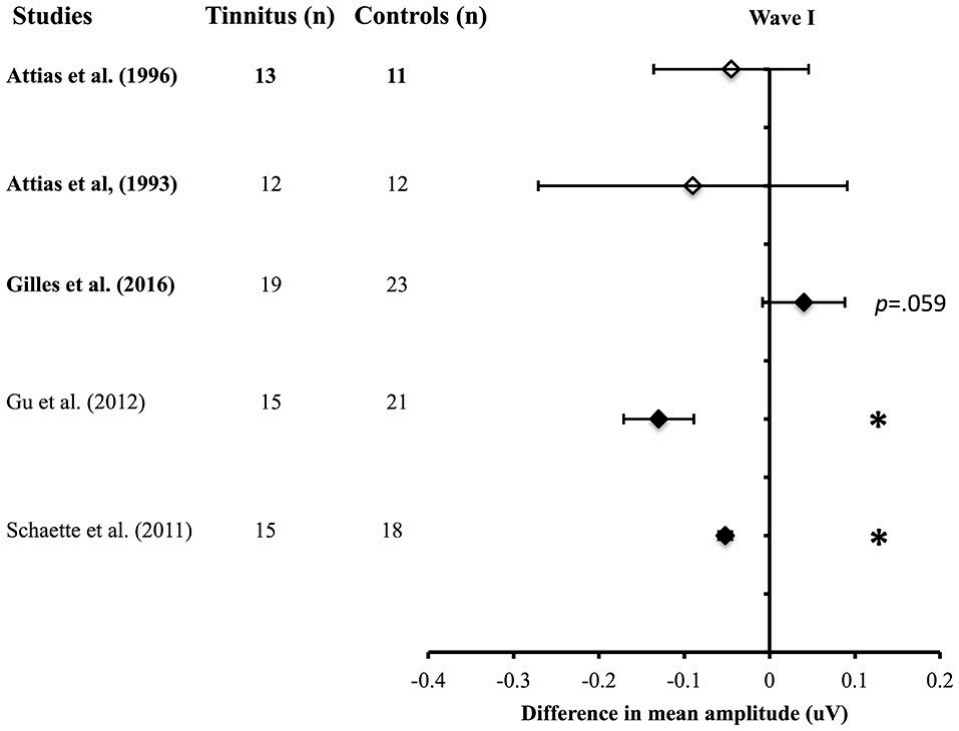
Meta-analysis (2) of the net difference in amplitudes for Wave I (95% CI) between tinnitus and control groups when matched for hearing status. Black diamonds represent studies with normal hearing participants and white diamonds studies with hearing loss participants. The studies in bold included only noise-induced tinnitus. ^*^Asterisks represent significant differences (*p* < 0.05).

For the mean amplitude differences, the second meta-analysis revealed that only two studies out of five found a significant reduction of wave I (see Figure 3). Two of the studies that did not report significant reduction of wave I amplitude tested noise-induced hearing loss participants with and without tinnitus (Figure 3, white diamonds). Gilles et al. (2016) reported a tendency of the wave I amplitude to be increased in the tinnitus group, although this did not reach significance (Figure 3).

## Discussion

The aim of the present scoping review was to investigate whether consistent ABR abnormalities are prevalent in populations with tinnitus. Although there is increasing interest in the use of ABRs for measuring auditory function in tinnitus individuals, the present scoping review found that the evidence of abnormalities within this population is sparse. Only 22 studies corresponding to the broad inclusion and exclusion criteria were found. Of these 22 studies, only 19 used control groups to make their comparisons. The present review unfortunately indicates that the tested tinnitus populations (i.e., *who*) are typically poorly defined across ABR studies as the vast majority did not report tinnitus etiology, assess and/or report the psychoacoustic properties of tinnitus, did not measure high frequency thresholds (above 8 kHz) and used various definitions of normal hearing and/or hearing loss. In regards to the methodology used (i.e., *how*), the ABR system, the type of transducer, the presentation level and the filtering strategies varied significantly across the studies. Still, the results of these studies (i.e., *what*) showed significant changes in amplitude and/or latency for high intensity stimulation levels as the current review did not assess low stimulation levels. In addition to this, longer latency and reduced amplitude of wave I for the normal hearing tinnitus group compared to hearing matched controls was consistently shown across numerous studies. Since high sound levels of stimulation were used in most studies, these results might indicate cochlear nerve fiber degeneration, a loss of neural synchrony, or both. These results will be further discussed by looking at the population characteristics, the various techniques and assessments, and the outcomes of the included studies. Based on these results, recommendations for future studies will be made as well as a description of the future direction of electrophysiology in tinnitus research.

### Heterogeneous population characteristics

One of the issues found by the current review is the poorly defined and undefined tinnitus population tested. Many of the studies reported the tinnitus from their test groups were either subjective or idiopathic. The vast majority did not report the tinnitus etiology at all. Only four studies chose noise-induced tinnitus as their sample population of which two included sensorineural hearing loss participants. Interestingly, none of the studies on noise-induced tinnitus reported any significant effects on wave latencies and amplitudes with the only exception being Attias et al. (1996) who found higher wave III amplitude in the tinnitus group compared to age and hearing matched controls. These null results contrast the findings by Gu et al. (2012) and Schaette and McAlpine (2011) showing reduced wave I amplitude in human tinnitus subjects, as well as animal studies showing decrease ABR wave I amplitudes at high stimulation levels after noise exposure without significant hearing threshold shifts (Kujawa and Liberman, 2006, 2009). Still, these human studies did not mention the etiology of the tinnitus nor did they classify their subjects as having noise-induced tinnitus. Conversely, the Kujawa and Liberman (2006, 2009) animal studies did not assess tinnitus. Thus, the direct link between ABR abnormalities obtained in noise-induced animals and humans is not completely elucidated. Indeed, a recent study investigating ABRs in a young adult cohorts with normal audiograms but exposed to noise, did not find any significant reductions of wave I (Prendergast et al., 2017). More so, a recent study on a young adult sample (early 20's) of noise-induced tinnitus found no differences in the amplitude and latency of any of the ABR waves (Gilles et al., 2016). In that study, the ABR was assessed only on a subgroup of tinnitus and controls subjects. Their participants were matched for age, sex, and hearing thresholds for pure tones of 1–4 kHz only. Since a measure of synaptopathy such as the AP/SP ratio of the electrocochleography and very high frequency thresholds have been shown to be correlated (Liberman et al., 2016), it is thus possible that the tinnitus group had better thresholds at very high frequencies (>8 kHz) than the controls, and less synaptopathy. Still, this would be very unlikely considering that tinnitus subjects usually display more hearing loss than controls for those high frequencies when matched for normal thresholds at conventional frequencies (Fournier and Hébert, 2013). One possible interpretation of these results is that it takes some time, maybe years, for the nerve fibers to degenerate and therefore to effect the ABR responses. Another possibility is that ABRs are not sensitive enough to reveal synaptopathy and/or that synaptopathy loss is not necessary to develop tinnitus. Also, differences across species have been noted in the development of synaptopathy (Prendergast et al., 2017): losses of cochlear synapses have been shown to be irreversible in rodents but not in guinea pigs (although their function remained abnormal) (Liu et al., 2012; Shi et al., 2013; Song et al., 2016). Thus, one has to be cautious when comparing results from different animal species and, even more cautious, when translating such results to human listeners.

Very few studies assessed ABR wave characteristics between tinnitus and controls with hearing loss. Considering that tinnitus is often associated with hearing loss and remains rare in individuals with normal hearing, why have so few studies assessed ABR abnormalities in tinnitus participants with significant hearing loss? It is well known that two of three individuals with hearing loss will go on to develop tinnitus (Hoffman and Reed, 2004). It is possible these studies purposefully avoided recruiting participants with hearing loss in order to prevent known confounding effects of hearing loss on the ABR. However, ABR abnormalities in individuals with hearing loss might nevertheless help reveal certain underlying neural mechanisms responsible for tinnitus generation. To date, the only reported significant effect in this specific population is higher wave III amplitude (Attias et al., 1996).

We conducted the first meta-analysis to demonstrate the effects of tinnitus within a large clinical population separated based on hearing loss. The advantage of such an approach is that the population is more comparable to what would be seen in a clinical setting, and the higher power, due to the large sample size, increases the chances of revealing tinnitus-related cofactors, such as hearing loss, whilst reducing the effects of random variables not related to tinnitus (i.e., gender, thickness of the scalp, transducer frequency response). This analysis shows increased latencies and reduced amplitudes for all three waves (I, III, and V) for tinnitus subjects compared to controls (Table 4) with hearing loss. However, these results must be interpreted cautiously as the number of subjects with tinnitus was five to 20 times higher than the number of controls depending on the wave. This imbalance of the number of subjects is the result of compiling all the data available from the entire yield of studies even though four studies did not report a control group. These ABR effects did not survive the second meta-analysis where only studies with matched control groups were used: the only two studies that used matched hearing loss control groups did not report any significant changes (Attias et al., 1993, 1996). It can be argued that the longer latencies and lower amplitudes found in the first meta-analysis may be the result of the compiled tinnitus group having a greater degree of hearing loss than controls (for amplitude: Sand and Saunte, 1994; for latency: Keith and Greville, 1987). The possibility of a gender and/or an aging bias could also account for the differences obtained in meta-analysis one.

The present review highlighted some variability in the criteria used to define normal hearing and an even larger variability in defining hearing loss. Future studies should define normal hearing as thresholds of less or equal to 15 dB HL minimally at all standard clinical frequencies thus from 250 to 8,000 Hz (Clark, 1981). More so, the measurements of high frequency thresholds (>8 kHz) need to be undertaken as significant threshold elevation for those frequencies (10–16 kHz) have been recently shown and interpreted as an early sign of synaptopathy in humans (Liberman et al., 2016). More so, high frequency hearing loss (>8 kHz) in tinnitus patients with conventional normal hearing (250– 8,000 Hz) have been reported (Fournier and Hébert, 2013; Vielsmeier et al., 2015). It is thus crucial to control for high frequency thresholds, at least up to 16 kHz, when comparing tinnitus subjects to controls in order to distinguish with confidence the presence of synaptopathy. Participants should be separated and grouped based on the presence of hearing loss. In addition to this, the degree (mild, moderate, severe, or profound), the origin (cochlear vs. neural), and the configuration (flat, high, or low frequency slope, notch) of the hearing loss should be clearly defined and reported.

One other recommendation is to recruit tinnitus participants based on tinnitus etiology (or report etiology) and/or psychoacoustic measurements in order to separate the test groups. This in turn might show ABR related patterns within each subgroup that would be otherwise masked by the heterogeneity of the sample. Few studies used characteristics of the tinnitus perception, such as pitch and loudness matching of the tinnitus percept or residual inhibition, as a means to separate various tinnitus subtypes. For instance, Noreña et al. (1999) classified the late auditory evoked potentials in three tinnitus subgroups based on their self-reported changes of tinnitus perception in relation to noise. They were classified as having decreased, increased, or unchanged tinnitus perception in the presence of noise. Based on this classification, they found that patients with decreased tinnitus perception in noise had greater intensity-dependence and longer N1 latency than the subgroup that reported increased tinnitus perception. Within the included ABR articles for this review, Maurizi et al. (1985) used residual inhibition (RI); a known phenomenon where a temporary reduction in the loudness or even disappearance of tinnitus follows the cessation of a masking noise, to stratify unilateral tinnitus into positive or no RI subgroups. They found wave I was prolonged for the positive RI group and wave V was prolonged for the no RI group of the tinnitus ear compared to the contralateral ear. They also performed ABR testing before and after treatment for each group. Interestingly, they found that after masking, the positive RI group's longer wave I latency had disappeared but the no RI group's wave V latency did not change. Stratification of the tinnitus test population based on psychoacoustic methods and added information on the tinnitus etiology would be crucial for future studies on auditory evoked potentials.

To note, only one study reported a potential adverse effect of the ABR on tinnitus subject. Indeed, Gu et al. (2012) reported that they could not complete the ABR assessment in 10 participants because they did not tolerate the stimulus intensity level. The co-occurrence of hyperacusis, which is defined as a hypersensivitity to moderate to loud sounds, and tinnitus have been shown to be very high (Hébert et al., 2004, 2013; Dauman and Bouscau-Faure, 2005). Still, the Gu and colleagues group is the only one to have reported that the hypersensitivity was detrimental for the assessment. From all the studies found in the current review, four measured hyperacusis in different ways within their sample: one used the Khalfa questionnaire (Gilles et al., 2016) and the others used loudness discomfort levels (LDL) (Gerken et al., 2001; Cartocci et al., 2012; Gu et al., 2012). It is not known whether hyperacusis was detrimental for the ABR assessment in those studies, as none reported it. Future studies should address the potential adverse effects of ABR testing such as discomfort or pain on tinnitus patients with and without hyperacusis. A potential cut-off on a hyperacusis questionnaire or on a psychophysical method such as LDL could be used to triage those participants for which the procedure is judged to be safe from those at risk of discomfort.

### Various techniques and assessments

Several techniques and assessments revealed by the review may have impacted the ABR results. One suspect issue occurs with the type of transducer. Out of the studies that reported the type of transducer used, 11 used various types of supra-auricular headphones while only four used insert earphones. According to Van Campen et al. (1992), insert earphones such as the ER-3A insert earphones can increase interaural attenuation, ambient noise attenuation, patient comfort, and eliminate ear canal collapse. Their study measured the acoustic output of TDH 39P, TDH 49P, and ER-3A inserts earphones on a KEMAR mannequin and used the same transducers for measuring click ABRs on normal hearing adults. One of the main differences they showed was that both TDH earphones had greater ringing than ER-3As for stimulus intensities down to 15 dB nHL. In addition to this, when tested on normal hearing adults, the insert earphones elicited a wave V that was significantly more delayed by 1.15 and 1 ms when stimulated at 40 dB nHL than the TDH earphones. Additionally, ER-3A earphones produced a significantly smaller wave I but similar wave V amplitude at 80 dB nHL than the TDH earphones, resulting in a greater V/I amplitude ratio. Given these differences, comparing data between insert and TDH earphones may be problematic.

Another potential issue comes from the frequency response of using various transducers with different response bandwidths. For example, the frequency response of an ER-3A earphone to a 500 Hz tone at 118.5 dB SPL is flat up to 4 kHz (E-A-R® Tone™ calibration specification sheet). This contrasts the Sennheiser HDA-200 headphones used by Gu et al. (2012) that was reported to have a bandwidth up to 8 kHz or the TDH 49 headphones that stimulate up to 7.1 kHz (Guest et al., 2016). Derived band measurements of the ABR to click stimuli show that wave I is mostly generated by characteristic frequencies above 2 kHz however wave V can be evoked by lower frequencies (Don and Eggermont, 1978; Abdala and Folsom, 1995). This may mean that the frequency response of the transducers used may influence the intensity of certain frequencies that may differ between studies. This variability may also contribute to the differences in latencies and amplitudes reported.

### Heterogeneous outcomes

The results found from the 22 studies were quite heterogeneous. For latencies, nine studies reported no change for any of the waves compared to nine who reported increased latency for waves I, V, and VII. Still, most well-controlled studies with appropriate matching procedures reported longer latencies for tinnitus compared to controls with wave I being the most consistently affected wave (Figure 2). A significant latency shift for all the three waves was found in Kehrle et al. (2008, 2016) and close to significance in Ikner and Hassen (1990) study. In these cases, the latency shift seen for waves III and V are not likely to be the result of the delayed wave I latency (due to neural damage) following through the other waves because the inter-peaks (I–V, III–V) were reported as abnormal in those same studies (see Table 3). These results might be related to a lack of central compensation in tinnitus individuals as suggested by Rüttiger et al. (2013).

Similar discordances were found within the amplitude data: four studies did not find any changes in amplitude for tinnitus, four reported decreased wave I, and either a decreased, an increased or even no modifications of the following amplitudes of waves III and V. Overall, only two of the five well-controlled studies reported decreased wave I amplitude. In addition to this, two well-controlled studies reported a higher V/I ratio (Kehrle et al., 2008; Nemati et al., 2014). More well-controlled studies are thus needed to clarify the presence of synaptopathy, as measured by wave I amplitude, in tinnitus patients. The large variability within the two studies with hearing loss tinnitus subjects prevent any conclusions that the trend towards lower wave I amplitude is due to an actual effect in tinnitus.

These mixed amplitude results may be related to the relative contribution of each type of nerve fibers (i.e., low-, medium-, and high-spontaneous rate) on the ABR signal. All the reviewed studies used high intensity stimuli that can be presumed to saturate the HSR fibers revealing potential difference linked only to LSR fibers. However, the specific contribution of each neural population (i.e., low-, medium-, and high- spontaneous rate) on the ABR waveform is not known. Substantial damage, for example to LSR fibers, may contribute to changes in the ABR amplitude in addition to the high spontaneous rate fibers. Bourien et al. (2014) have recently demonstrated that LSR fibers might have a negligible contribution to wave I ABR by measuring the compound action potential after selective damage to the LSR auditory nerve fibers of gerbils. They suggested that wave I reduction might be the result of damage to medium spontaneous rate fibers, which are usually mixed with LSR fibers in previous studies. It is also possible that damage to the LSR and MSR fibers varies across the length of the basilar membrane in such a way that the regions corresponding to certain frequencies have less damage than other areas. One way to target regions specifically affected by synaptopathy is to use specific frequencies or tone bursts, however narrowing the stimulus to include fewer frequencies may further reduce the number of responding fibers. For example, Gerken et al. (2001) used 10 tone bursts (1, 1.5, 2, 2.5, 3, 3.5, 4, 5, 6, and 8 kHz) at a level of 112 dB SPL to elicit the ABR in a tinnitus and non-tinnitus population with and without hearing loss. They found no significant differences for the ABR amplitude and latencies (Wave I, III, V) of the tinnitus group compared to the non-tinnitus subjects. Still, they did not use any matching procedures to compose their groups and included only nine tinnitus subjects with hearing loss. It may be interesting to replicate the Gerken et al. (2001) study on normal hearing populations (for all frequencies up to 16 kHz) with and without tinnitus, and with appropriate matching procedures (gender, age, and hearing status) for different intensity levels, for different frequencies (from low to high, including tinnitus pitch) in order to compare the response of the HSR, MSR, and LSR fibers.

Since the search for this review was conducted, two more articles on ABR and tinnitus populations have been published: Ravikumar and Murthy (2016) and Guest et al. (2016). Both studies compared tinnitus populations with normal hearing to normal hearing matched controls. Normal hearing was not defined in the former, and was defined as pure tone thresholds of ≤20 dB HL at 0.25–8 kHz for the latter. Latencies of wave I, III, and V were significantly (*p* = 0.05) prolonged (Ravikumar and Murthy, 2016) and wave V amplitude was higher but not significant (Guest et al., 2016). The latter study also reported no differences in the amplitude of wave I and found there was no correlation between the amplitude of wave I and history of noise exposure.

### Recommendations for future studies on ABR

Clear and simple recommendations for future ABR investigations on tinnitus can be determined from these findings with the aim of improving future reviews on the subject, showing more reliable evidence of tinnitus, and making it easier to replicate previous studies. Most imperatively, it is highly encouraged that researchers report all the data collected including latencies and amplitudes of all the waves in a format that is suitable for meta-analysis. The meta-analysis of the current study was difficult particularly when latency or amplitude data was left unreported. From 22 studies found on ABR investigations of tinnitus in humans, only 10 studies could be used for the second meta-analysis to compare the mean difference of latencies between tinnitus and controls. Unfortunately, this represented <50% of all the studies found. For amplitude, even fewer studies were retained (*n* = 5), which represents <25% of all the studies. The mean and standard deviation of the latencies and amplitudes of the waves found within their paradigm for the tinnitus and control groups should be reported separately. Negative and non-significant results should always be reported in a similar fashion.

Secondly, all future ABR protocols should at least include control groups matched for gender, age and hearing status for sufficient control over these covariables. As mentioned previously, the two studies displaying the greatest variability for latencies and amplitudes in the second meta-analysis (Attias et al., 1993, 1996) did not match for gender between groups. Still, when comparing more recent studies to older ones, there appears to be a clear trend toward the use of more restrictive matching procedures. It is further suggested that hearing be assessed and matched for frequencies up to at least 16 kHz between groups. Studies should recruit participants with similar tinnitus etiologies (e.g., noise trauma) and include psychoacoustic measurements such as pitch and loudness matching, minimum masking level and residual inhibition. Future research should also consider separating participants into narrower age bands or at least separate younger and older subjects into two different groups. The two studies reporting reduced wave I in tinnitus (Schaette and McAlpine, 2011; Gu et al., 2012) tested participants approximately 10 years older on average than the study of Gilles et al. (2016) which included only participants below the age of 30. The absence of synaptopathy using wave I amplitude has also been reported in a study on noise-exposed young adults (mean age of 23, ranging from 18 to 36 years old), however tinnitus was not assessed (Prendergast et al., 2017). It is thus recommended that an age cut-off around 30 years old be used for future work. A sample size of at least 30 subjects per group is also recommended in order to reduce variability of the measures and to increase statistical power. Regarding the technical aspects of ABR measurement, insert headphones are preferred over circumauricular ones in order to optimize interaural attenuation, ambient noise attenuation, and to reduce the risk of ear canal collapsing. The frequency response of the transducer should also include as many frequencies above 2 kHz as possible.

### Future directions

Since the key publication of animal research demonstrating evidence of cochlear synaptopathy after noise exposure (Kujawa and Liberman, 2006, 2009), there has been a growing interest in improving ABR measurements in humans. Reliable ABR waveforms can sometimes be difficult to obtain mostly because of high inter-subject variability due to factors such as small signal to noise ratios, head shape, sex, as well as the various methodological concerns described above. Many research groups have attempted to address these issues by either improving the methodology of the click or tone-burst ABR method or by proposing new methods of assessment. For example, one study used an electrode placed on the tympanic membrane (TM) in order to improve the signal to noise ratio (Stamper and Johnson, 2015). Using a similar electrode tip on the TM, another group used electrocochleography instead of ABR to show significant differences in the SP/AP ratio between high and low noise exposure risk groups of participants (Liberman et al., 2016). This finding still needs to be replicated, but electrocochleography could potentially become a standard measure of synaptopathy instead of the classical ABR. Another group showed delayed wave V ABRs when responding to clicks in background noise as evidence of the presence of synaptopathy in animals and humans (Mehraei et al., 2016). The use of envelope following responses (EFR) with amplitude-modulated tones in notched noise with varying modulation depth have also shown deficits that are consistent with synaptopathy (Bharadwaj et al., 2015). All these new techniques could easily be applied to tinnitus participants. This in turn can bring new insight on a possible role, if any, played by cochlear synaptopathy in the generation of tinnitus. More so, the application of a paradigm to desynchronize neural activity may help reveal potential tinnitus mechanisms. Indeed, when click-rate is increased, the nerve fibers appear to have more difficulty synchronizing their discharge to the stimulus, resulting in smaller ABR amplitudes and prolonged wave V latencies (Konrad-Martin et al., 2012). Higher synchronous activity at higher levels of the auditory system related to tinnitus might thus only be revealed when using high click-rates.

Finally, ABRs have more recently been used not only to understand the pathophysiology of tinnitus but also objectify its presence in individuals. The gap-in-noise ABR (or GIN-ABR) has been used in animal subjects with different background noise frequencies before and after tinnitus induction by salicylate (Lowe and Walton, 2015). Using this method, they found a significant reduction in gap detection after salicylate treatment for only the 16 kHz background noise condition. The authors concluded that since salicylate is known to produce a 16 kHz tinnitus percept that appears to fill the gap, the GIN-ABR may be effective for objectifying the presence of tinnitus in animals. This in turn may be a promising new avenue for future auditory brainstem research applied to humans with tinnitus.

## Author contributions

All the authors contributed to this work. VM and AK provided the original conception and design of the study. VM and PF worked on data acquisition, analysis, and interpretation. They both wrote the manuscript. DB, AN, and AK provided intellectual feedback and revised the content of several previous versions of the manuscript. All authors (VM, PF, DB, AN, and AK) approved the final version of the manuscript.

### Conflict of interest statement

The authors declare that the research was conducted in the absence of any commercial or financial relationships that could be construed as a potential conflict of interest. The reviewer NKE and handling Editor declared their shared affiliation, and the handling Editor states that the process nevertheless met the standards of a fair and objective review.

## References

[B1] Abdala C. Folsom R. C. (1995). Frequency contribution to the click-evoked auditory-brain-stem response in human adults and infants. J. Acoust. Soc. Am. 97, 2394–2404. 10.1121/1.411961 7714257

[B2] Arksey H. O'Malley L. (2005). Scoping studies: towards a methodological framework. Int. J. Soc. Res. Methodol. 8, 19–32. 10.1080/1364557032000119616

[B3] Attias J. Pratt H. Reshef I. Bresloff I. Horowitz G. Polyakov A.. (1996). Detailed analysis of auditory brainstem responses in patients with noise-induced tinnitus. Audiology 35, 259–270. 10.3109/00206099609071946 8937658

[B4] Attias J. Urbach D. Gold S. Shemesh Z. (1993). Auditory event related potentials in chronic tinnitus patients with noise induced hearing loss. Hear. Res. 71, 106–113. 10.1016/0378-5955(93)90026-W 8113129

[B5] Barnea G. Attias J. Gold S. Shahar A. (1990). Tinnitus with normal hearing sensitivity: extended high-frequency audiometry and auditory-nerve brain-stem-evoked responses. Audiology 29, 36–45. 10.3109/00206099009081644 2310352

[B6] Bauch C. D. Olsen W. O. Pool A. F. (1996). ABR indices: sensitivity, specificity, and tumor size. Am. J. Audiol. 5, 97–104. 10.1044/1059-0889.0501.97

[B7] Bayar N. Böke B. Turan E. Belgin E. (2001). Efficacy of amitriptyline in the treatment of subjective tinnitus. J. Otolaryngol. 30, 300–303. 10.2310/7070.2001.19597 11771024

[B8] Berliner K. Shelton C. Hitselberger W. (1992). Acoustic tumors: effect of surgical removal on tinnitus. Otol. Neurotol. 13, 13–17. 10.1097/00129492-199201000-00005 1598977

[B9] Bharadwaj H. M. Masud S. Mehraei G. Verhulst S. Shinn-Cunningham B. G. (2015). Individual differences reveal correlates of hidden hearing deficits. J. Neurosci. 35, 2161–2172. 10.1523/JNEUROSCI.3915-14.2015 25653371 PMC4402332

[B10] Bourien J. Tang Y. Batrel C. Huet A. Lenoir M. Ladrech S.. (2014). Contribution of auditory nerve fibers to compound action potential of the auditory nerve. J. Neurophysiol. 112, 1025–1039. 10.1152/jn.00738.2013 24848461

[B11] Burkard R. Secor C. (2002). Overview of auditory evoked potentials, in Handbook of Clinical Audiology, ed Julet T. L. (Baltimore, MD: Lippincott Williams and Wilkins), 233–248.

[B12] Cartocci G. Attanasio G. Fattapposta F. Locuratolo N. Mannarelli D. Filipo R. (2012). An electrophysiological approach to tinnitus interpretation. Int. Tinnitus J. 17, 152–157. 10.5935/0946-5448.20120027 24333887

[B13] Chalak S. Kale A. Deshpande V. K. Biswas D. A. (2013). Establishment of normative data for monaural recordings of auditory brainstem response and its application in screening patients with hearing loss: a cohort study. J. Clin. Diagn. Res. 7, 2677–2679. 10.7860/jcdr/2013/6768.3730 24551609 PMC3919392

[B14] Clark J. (1981). Uses and abuses of hearing loss. ASHA 23, 493–500. 7052898

[B15] Dauman R. Bouscau-Faure F. (2005). Assessment and amelioration of hyperacusis in tinnitus patients. Acta Otolaryngol. 125, 503–509. 10.1080/00016480510027565 16092541

[B16] De Lavernhe-Lemaire M. C. Beutter P. (1989). Potentiels évoqués auditifs dans la distinction entre acouphènes périphériques et centraux. Arch. Int. Physiol. Biochim. 97, 135–144. 10.3109/13813458909104533 2476091

[B17] De Lavernhe-Lemaire M. C. Beutter P. (1990). Modifications des amplitudes des potentiels évoques auditifs précoces observées dans les acouphènes. Arch. Int. Physiol. Biochim. 98, 403–409. 10.3109/13813459009114002 1705779

[B18] De Ridder D. Vanneste S. Langguth B. Llinas R. (2015). Thalamocortical dysrhythmia: a theoretical update in tinnitus. Front. Neurol. 6:124. 10.3389/fneur.2015.00124 26106362 PMC4460809

[B19] Don M. Eggermont J. J. (1978). Analysis of the click-evoked brainstem potentials in man using high-pass noise masking. J. Acoust. Soc. Am. 63, 1084–1092. 10.1121/1.381816 649867

[B20] Don M. Ponton C. W. Eggermont J. J. Kwong B. (1998). The effects of sensory hearing loss on cochlear filter times estimated from auditory brainstem response latencies. J. Acoust. Soc. Am. 104, 2280–2289. 10.1121/1.423741 10491692

[B21] Eggermont J. J. (1984). Tinnitus: some thoughts about its origin. J. Laryngol. Otol. 98, 31–37. 10.1017/S17551463000900896693802

[B22] Fournier P. Hébert S. (2013). Gap detection deficits in humans with tinnitus as assessed with the acoustic startle paradigm: does tinnitus fill in the gap? Hear. Res. 295, 16–23. 10.1016/j.heares.2012.05.011 22688322

[B23] Gerken G. M. Hesse P. S. Wiorkowski J. J. (2001). Auditory evoked responses in control subjects and in patients with problem-tinnitus. Hear. Res. 157, 52–64. 10.1016/S0378-5955(01)00277-5 11470185

[B24] Gilles A. Schlee W. Rabau S. Wouters K. Fransen E. Van de Heyning P. (2016). Decreased speech-in-noise understanding in young adults with tinnitus. Front. Neurosci. 10:288. 10.3389/fnins.2016.00288 27445661 PMC4923253

[B25] Giraudet F. Avan P. (2012). Auditory neuropathies. Curr. Opin. Neurol. 25, 50–56. 10.1097/WCO.0b013e32834f0351 22185903

[B26] Gopal K. V. Thomas B. P. Mao D. Lu H. (2015). Efficacy of carnitine in treatment of tinnitus: evidence from audiological and MRI measures—a case study. J. Am. Acad. Audiol. 26, 311–324. 10.3766/jaaa.26.3.10 25751698

[B27] Gu J. W. Herrmann B. S. Levine R. A. Melcher J. R. (2012). Brainstem auditory evoked potentials suggest a role for the ventral cochlear nucleus in tinnitus. J. Assoc. Res. Otolaryngol. 13, 819–833. 10.1007/s10162-012-0344-1 22869301 PMC3505586

[B28] Guest H. Munro K. J. Prendergast G. Howe S. Plack C. J. (2016). Tinnitus with a normal audiogram: relation to noise exposure but no evidence for cochlear synaptopathy. Hear. Res. 344, 265–274. 10.1016/j.heares.2016.12.002 27964937 PMC5256478

[B29] Hébert S. Fournier P. Noreña A. (2013). The auditory sensitivity is increased in tinnitus ears. J. Neurosci. 33, 2356–2364. 10.1523/JNEUROSCI.3461-12.2013 23392665 PMC6619157

[B30] Hébert S. Paiement P. Lupien S. J. (2004). A physiological correlate for the intolerance to both internal and external sounds. Hear. Res. 190, 1–9. 10.1016/S0378-5955(04)00021-8 15051125

[B31] Henry J. A. Roberts L. E. Caspary D. M. Theodoroff S. M. Salvi R. J. (2014). Underlying mechanisms of tinnitus: review and clinical implications. J. Am. Acad. Audiol. 25, 5–22. 10.3766/jaaa.25.1.2 24622858 PMC5063499

[B32] Hickox A. E. Liberman M. C. (2014). Is noise-induced cochlear neuropathy key to the generation of hyperacusis or tinnitus? J. Neurophysiol. 111, 552–564. 10.1152/jn.00184.2013 24198321 PMC3921399

[B33] Hoffman H. Reed G. (2004). Epidemiology of tinnitus, in Tinnitus: Theory and Management, ed Snow J. B. (Lewiston, NY: BC Decker), 16–41.

[B34] House J. W. Brackmann D. E. (1981). Tinnitus: surgical treatment, in Ciba Foundation Symposium 85: Tinnitus, eds Evered D. Lawrens G. (London: Pitman), 204–212. 10.1002/9780470720677.ch12 6915835

[B35] Hultcrantz M. Simonoska R. Stenberg A. E. (2006). Estrogen and hearing: a summary of recent investigations. Acta Otolaryngol. 126, 10–14. 10.1080/00016480510038617 16308248

[B36] Hyde M. L. Riko K. Malizia K. (1990). Audiometric accuracy of the click ABR in infants at risk for hearing loss. J. Am. Acad. Audiol. 1, 59–66. 2132587

[B37] Ikner C. L. Hassen A. H. (1990). The effect of tinnitus on ABR latencies. Ear Hear. 11, 16–20. 10.1097/00003446-199002000-00005 2307297

[B38] Jewett D. Romano M. Williston J. (1970). Human auditory evoked potentials: possible brain stem components detected on the scalp. Science 167, 1517–1518. 10.1126/science.167.3924.1517 5415287

[B39] Jewett D. Williston J. S. (1971). Auditory-evoked far fields averaged from the scalp of humans. Brain 94, 681–696. 10.1093/brain/94.4.681 5132966

[B40] Kehrle H. M. Granjeiro R. C. Sampaio A. L. L. Bezerra R. Almeida V. F. Oliveira C. A. (2008). Comparison of auditory brainstem response results in normal-hearing patients with and without tinnitus. Arch. Otolaryngol. Head Neck Surg. 134, 647–651. 10.1001/archotol.134.6.647 18559734

[B41] Kehrle H. M. Sampaio A. L. Granjeiro R. C. De Oliveira T. S. Oliveira C. A. (2016). Tinnitus annoyance in normal-hearing individuals: correlation with depression and anxiety. Ann. Otol. Rhinol. Laryngol. 125, 185–194. 10.1177/0003489415606445 26424781

[B42] Keith W. J. Greville K. A. (1987). Effects of audiometric configuration on the auditory brain stem response. Ear Hear. 8, 49–55. 10.1097/00003446-198702000-00009 3556811

[B43] Khangura S. Konnyu K. Cushman R. Grimshaw J. Moher D. (2012). Evidence summaries: the evolution of a rapid review approach. Syst. Rev. 1:10. 10.1186/2046-4053-1-10 22587960 PMC3351736

[B44] Kim S. I. Kim M. G. Kim S. S. Byun J. Y. Park M. S. Yeo S. G. (2016). Evaluation of tinnitus patients by audiometric configuration. Otolaryngol. Head Neck Surg. 37, 1–5. 10.1016/j.amjoto.2015.08.009 26700250

[B45] Konrad-Martin D. Dille M. F. McMillan G. Griest S. McDermott D. Fausti S. A.. (2012). Age-related changes in the auditory brainstem response. J. Am. Acad. Audiol. 23, 18–35. 10.3766/jaaa.23.1.3 22284838 PMC5549623

[B46] Kotlarz J. P. Eby T. L. Borton T. E. (1992). Analysis of the efficiency of retrocochlear screening. Laryngoscope 102, 1108–1112. 10.1288/00005537-199210000-00004 1405961

[B47] Kujawa S. G. Liberman M. C. (2006). Acceleration of age-related hearing loss by early noise exposure: evidence of a misspent youth. J. Neurosci. 26, 2115–2123. 10.1523/JNEUROSCI.4985-05.2006 16481444 PMC1855187

[B48] Kujawa S. G. Liberman M. C. (2009). Adding insult to injury: cochlear nerve degeneration after “temporary” noise-induced hearing loss. J. Neurosci. 29, 14077–14085. 10.1523/JNEUROSCI.2845-09.2009 19906956 PMC2812055

[B49] Lemaire M. C. Beutter P. (1995). Brainstem auditory evoked responses in patients with tinnitus. Audiology 34, 287–300. 10.3109/00206099509071919 8833309

[B50] Levac D. Colquhoun H. O'Brien K. K. (2010). Scoping studies: advancing the methodology. Implement. Sci. 5:69. 10.1186/1748-5908-5-69 20854677 PMC2954944

[B51] Levine R. A. (1999). Somatic (craniocervical) tinnitus and the dorsal cochlear nucleus hypothesis. Am. J. Otolaryngol. 6, 351–362. 10.1016/S0196-0709(99)90074-1 10609479

[B52] Lewis J. D. Kopun J. Neely S. T. Schmid K. K. Gorga M. P. (2015). Tone-burst auditory brainstem response wave V latencies in normal-hearing and hearing-impaired ears. J. Acoust. Soc. Am. 138, 3210–3219. 10.1121/1.4935516 26627795 PMC4662677

[B53] Liberman M. C. Epstein M. J. Cleveland S. S. Wang H. Maison S. F. (2016). Toward a differential diagnosis of hidden hearing loss in humans. PLoS ONE 11:e0162726. 10.1371/journal.pone.0162726 27618300 PMC5019483

[B54] Liberman M. C. Kujawa S. G. (2017). Cochlear synaptopathy in acquired sensorineural hearing loss: manifestations and mechanisms. Hear. Res. 349, 138–147. 10.1016/j.heares.2017.01.003 28087419 PMC5438769

[B55] Liu L. Wang H. Shi L. Almuklass A. He T. Aiken S.. (2012). Silent damage of noise on cochlear afferent innervation in guinea pigs and the impact on temporal processing. PLoS ONE 7:e49550. 10.1371/journal.pone.0049550 23185359 PMC3504112

[B56] Lowe A. S. Walton J. P. (2015). Alterations in peripheral and central components of the auditory brainstem response: a neural assay of tinnitus. PLoS ONE 10:e0117228. 10.1371/journal.pone.0117228 25695496 PMC4335042

[B57] Mahmoudian S. Lenarz M. Esser K.-H. Salamat B. Alaeddini F. Dengler R.. (2013). Alterations in early auditory evoked potentials and brainstem transmission time associated with tinnitus residual inhibition induced by auditory electrical stimulation. Int. Tinnitus J. 18, 63–74. 10.5935/0946-5448.20130009 24995901

[B58] Maurizi M. Ottaviani F. Paludetti G. Almadori G. Tassoni A. (1985). Contribution to the differentiation of peripheral versus central tinnitus via auditory brain stem response evaluation. Audiology 24, 207–216. 10.3109/00206098509070104 4004647

[B59] McKee G. J. Stephens S. D. G. (1992). An investigation of normally hearing subjects with tinnitus. Audiology 31, 313–317. 10.3109/00206099209072919 1492815

[B60] Mehraei G. Hickox A. E. Bharadwaj H. M. Goldberg H. Verhulst S. Liberman M. C.. (2016). Auditory brainstem response latency in noise as a marker of cochlear synaptopathy. J. Neurosci. 36, 3755–3764. 10.1523/JNEUROSCI.4460-15.2016 27030760 PMC4812134

[B61] Melcher J. R. Kiang N. Y. (1996). Generators of the brainstem auditory evoked potential in cat III: identified cell populations. Hear. Res. 93, 52–71. 10.1016/0378-5955(95)00200-6 8735068

[B62] Milicic D. Alcada M. N. (1999). A tinnitus objectivization: how we do it. Int Tinnitus J. 5, 5–15. 10753410

[B63] Moeller A. R. (1984). Pathophysiology of tinnitus. Ann. Otol. Rhinol. Laryngol. 93, 39–44. 10.1177/000348948409300110 6367601

[B64] Moher D. Liberati A. Tetzlaff J. Altman D. G. (2009). Academia and clinic annals of internal medicine preferred reporting items for systematic reviews and meta-analyses: the PRISMA statement. Ann. Intern. Med. 151, 264–269. 10.7326/0003-4819-151-4-200908180-00135 19622511

[B65] Nemati S. Habibi A. F. Panahi R. Pastadast M. (2014). Cochlear and brainstem audiologic findings in normal hearing tinnitus subjects in comparison with non-tinnitus control group. Acta Med. Iran. 51, 822–826. 25415814

[B66] Noreña A. Cransac H. Chéry-Croze S. (1999). Towards an objectification by classification of tinnitus. Clin. Neurophysiol. 110, 666–675. 10378736 10.1016/s1388-2457(98)00034-0

[B67] Noreña A. J. (2011). An integrative model of tinnitus based on a central gain controlling neural sensitivity. Neurosci. Biobehav. Rev. 35, 1089–1109. 10.1016/j.neubiorev.2010.11.003 21094182

[B68] Prendergast G. Guest H. Munro K. J. Kluk K. Léger A. Hall D. A.. (2017). Effects of noise exposure on young adults with normal audiograms I: electrophysiology. Hear. Res. 344, 68–81. 10.1016/j.heares.2016.10.028 27816499 PMC5256477

[B69] Ravikumar G. Murthy V. A. (2016). A study of brainstem auditory evoked responses in normal hearing patients with tinnitus. Indian J. Otolaryngol. Head Neck Surg. 68, 429–433. 10.1007/s12070-015-0917-5 27833867 PMC5083643

[B70] Rosenhall U. Axelsson A. (1994). Auditory brainstem response latencies in patients with tinnitus. Scand. Audiol. 24, 97–100. 10.3109/01050399509047521 7660061

[B71] Rupa V. Job A. George M. Rajshekhar V. (2003). Cost-effective initial screening for vestibular schwannoma: auditory brainstem response or magnetic resonance imaging? Otolaryngol. Head Neck Surg. 128, 823–828. 10.1016/S0194-5998(03)00358-9 12825033

[B72] Rüttiger L. Singer W. Panford-Walsh R. Matsumoto M. Lee S. C. Zuccotti A.. (2013). The reduced cochlear output and the failure to adapt the central auditory response causes tinnitus in noise exposed rats. PLoS ONE 8:e57247. 10.1371/journal.pone.0057247 23516401 PMC3596376

[B73] Sand T. Saunte C. (1994). ABR amplitude and dispersion variables: relation to audiogram shape and click polarity. Scand. Audiol. 23, 7–12. 10.3109/01050399409047482 8184286

[B74] Santos-Filha V. Samelli A. Matas C. (2014). Noise-induced tinnitus: auditory evoked potential in symptomatic and asymptomatic patients. Clinics 69, 487–490. 10.6061/clinics/2014(07)08 25029581 PMC4081887

[B75] Schaette R. McAlpine D. (2011). Tinnitus with a normal audiogram: physiological evidence for hidden hearing loss and computational model. J. Neurosci. 31, 13452–13457. 10.1523/JNEUROSCI.2156-11.2011 21940438 PMC6623281

[B76] Shi L. Liu L. He T. Guo X. Yu Z. Yin S.. (2013). Ribbon synapse plasticity in the cochleae of Guinea pigs after noise-induced silent damage. PLoS ONE 8:e81566. 10.1371/journal.pone.0081566 24349090 PMC3857186

[B77] Shulman A. Seitz M. R. (1981). Central tinnitus- diagnosis and treatment. Observations simultaneous binaural auditory brain responses with monaural stimulation in the tinnitus patient. Laryngoscope 91, 2025–2036. 10.1288/00005537-198112000-00005 7321723

[B78] Singh S. Munjal S. K. Panda N. K. Munjal K. S. Panda K. N. Munjal S. K.. (2011). Comparison of auditory electrophysiological responses in normal-hearing patients with and without tinnitus. J. Laryngol. Otol. 125, 668–672. 10.1017/S0022215111000569 21554838

[B79] Song Q. Shen P. Li X. Shi L. Liu L. Wang J.. (2016). Coding deficits in hidden hearing loss induced by noise: the nature and impacts. Sci. Rep. 6:25200. 10.1038/srep25200 27117978 PMC4846864

[B80] Stamper G. C. Johnson T. A. (2015). Auditory function in normal-hearing, noise-exposed human ears. Ear Hear. 36, 172–184. 10.1097/AUD.0000000000000107 25350405 PMC4374361

[B81] Starr A. Picton T. W. Sininger Y. Hood L. J. Berlin C. I. (1996). Auditory neuropathy. Brain 119, 741–753. 10.1093/brain/119.3.741 8673487

[B82] Stouffer J. L. Tyler R. S. (1990). Characterisation of tinnitus by tinnitus patients. J. Speech Hear. Disord. 55, 439–453. 10.1044/jshd.5503.439 2381186

[B83] Tyler R. Coelho C. Tao P. Ji H. (2008). Identifying tinnitus subgroups with cluster analysis. Am. J. Audiol. 17, S176–S184. 10.1044/1059-0889(2008/07-0044) 19056922 PMC2668860

[B84] Van Campen L. E. Sammeth C. A. Hall J. W. Peek B. F. (1992). Comparison of Etymotic insert and TDH supra-aural earphones in auditory brainstem response measurement. J. Am. Acad. Audiol. 3, 315–323. 1421467

[B85] Vielsmeier V. Lehner A. Strutz J. Steffens T. Kreuzer P. M. Schecklmann M.. (2015). The relevance of the high frequency audiometry in tinnitus patients with normal hearing in conventional pure-tone audiometry. Biomed. Res. Int. 2015:302515. 10.1155/2015/302515 26583098 PMC4637018

[B86] Watson D. R. (1996). The effects of cochlear hearing loss. Audiology 35, 246–258. 10.3109/00206099609071945a 8937657

[B87] Wilson D. F. Hodgson R. S. Gustafson M. F. Hogue S. Mills L. (1992). The sensitivity of auditory brainstem response testing in small acoustic neuromas. Laryngoscope 102, 961–964. 10.1288/00005537-199209000-00001 1518359

[B88] Yu H. Patil K. V. Han C. Fabella B. Canlon B. Someya S.. (2016). GLAST deficiency in mice exacerbates gap detection deficits in a model of salicylate-induced tinnitus. Front. Behav. Neurosci. 10:158. 10.3389/fnbeh.2016.00158 27582696 PMC4987341

