# Supplementary material for: Auditory Brainstem Responses in Tinnitus: A Review of Who, How, and What?
Source: Front Aging Neurosci. 2017 Jul 21;9:237. doi: 10.3389/fnagi.2017.00237 (PMC5519563; doi:10.3389/fnagi.2017.00237)
Supplement: Supplementary file 1 [file Table1.DOCX]

Supplementary Material

Auditory brainstem responses in tinnitus: a review of Whom, How and What?

**Victoria Milloy* ^1^, Philippe Fournier ^2^, Daniel Benoit ^1^, Arnaud Noreña ^2^, Amineh Koravand ^1^**

*** Correspondence:** Victoria Milloy: vmilloy@uottawa.ca

**Supplementary Table 1:** Search Strategy

| **PubMed** | **MedLine** | **Embase** | **PsycINFO** | **CINAHL** | **ProQuest Dissertations & Theses Global** | **Conference Papers Index** |
| --- | --- | --- | --- | --- | --- | --- |
| ((evoked potentials, auditory, brain stem[MeSH Terms]) OR (brainstem response*) OR (brainstem potential*) OR (brain stem response*) OR (brain stem potential*) OR (ABR*) OR (BAER*) OR (BSER*)) AND ((tinnit*) OR (ear* AND (buzz* OR ring* OR roar* OR click* OR pulsat* OR pulse*)) OR Tinnitus[MESH]) | 1. Tinnitus/  2. tinnit*.tw.  3. (ear* and (buzz* or ring* or roar* or click* or pulsat* or pulse*)).tw.  4. 1 or 2 or 3  5. Evoked Potentials, Auditory, Brain Stem/  6. (brainstem adj3 response*).tw.  7. (brainstem adj3 potential*).tw.  8. (brain stem adj3 response*).tw.  9. (brain stem adj3 potential*).tw.  10. ABR*.tw.  11. BAER*.tw.  12. BSER*.tw.  13. 5 or 6 or 7 or 8 or 9 or 10 or 11 or 12  14. 4 and 13 | 1. Tinnitus/  2. tinnit*.tw.  3. (ear* and (buzz* or ring* or roar* or click* or pulsat* or pulse*)).tw.  4. 1 or 2 or 3  5. (brainstem adj3 response*).tw.  6. (brainstem adj3 potential*).tw.  7. (brain stem adj3 response*).tw.  8. (brain stem adj3 potential*).tw.  9. ABR*.tw.  10. BAER*.tw.  11. BSER*.tw.  12. evoked brain stem auditory response/ or brain stem response/ or evoked brain stem response/  13. 5 or 6 or 7 or 8 or 9 or 10 or 11 or 12  14. 4 and 13 | 1. Tinnitus/  2. tinnit*.tw.  3. (ear* and (buzz* or ring* or roar* or click* or pulsat* or pulse*)).tw.  4. 1 or 2 or 3  5. (brainstem adj3 response*).tw.  6. (brainstem adj3 potential*).tw.  7. (brain stem adj3 response*).tw.  8. (brain stem adj3 potential*).tw.  9. ABR*.tw.  10. BAER*.tw.  11. BSER*.tw.  12. auditory evoked potentials/  13. 5 or 6 or 7 or 8 or 9 or 10 or 11 or 12  14. 4 and 13 | S1. (MH "Tinnitus")  S2. tinnit*  S3. (buzz* OR ring* OR roar* OR click* OR pulsat* OR pulse*) AND ear*  S4. S1 OR S2 OR S3  S5. (MH "Evoked Potentials, Auditory, Brainstem")  S6. brainstem N3 response*  S7. brainstem N3 potential*  S8. brain stem N3 response*  S9. brain stem N3 potential*  S10. ABR*  S11. BAER*  S12. BSER*  S13. S5 OR S6 OR S7 OR S8 OR S9 OR S10 OR S11 OR S12  S14. S4 AND S13 | (all(tinnitus) OR all(tinnit*)) AND (all((evoked OR auditory) NEAR/3 potentials NEAR/3 (brainstem OR "brain stem")) OR all((brainstem NEAR/3 response* OR brainstem NEAR/3 potential*)) OR all((brain stem NEAR/3 response* OR brain stem NEAR/3 response*)) OR all((brain stem NEAR/3 potential* OR ABR)) OR all((BAER OR BSER))) | ((tinnitus) OR (tinnit*)) AND (((evoked OR auditory) NEAR/3 potentials NEAR/3 (brainstem OR "brain stem")) OR ((brainstem NEAR/3 response* OR brainstem NEAR/3 potential*)) OR ((brain stem NEAR/3 response* OR brain stem NEAR/3 response*)) OR ((brain stem NEAR/3 potential* OR ABR)) OR ((BAER OR BSER))) |
