# Supplementary material for: Auditory Brainstem Responses in Tinnitus: A Review of Who, How, and What?
Source: Front Aging Neurosci. 2017 Jul 21;9:237. doi: 10.3389/fnagi.2017.00237 (PMC5519563; doi:10.3389/fnagi.2017.00237)
Supplement: Supplementary file 2 [file Table2.DOCX]

Supplementary Material

Auditory brainstem responses in tinnitus: a review of Whom, How and What?

**Victoria Milloy* ^1^, Philippe Fournier ^2^, Daniel Benoit ^1^, Arnaud Noreña ^2^, Amineh Koravand ^1^**

*** Correspondence:** Victoria Milloy: vmilloy@uottawa.ca

**Supplementary Table 2:** Quantitative meta-analysis 1 table summarizing number of subjects, and mean latency and amplitude values for the tinnitus and control groups of each study.

| **Study name** | **Subjects** | | **Latency (Tinnitus)** | | | **Amplitude (Tinnitus)** | | | **Latency (Controls)** | | | **Amplitude (Controls)** | | |
| --- | --- | --- | --- | --- | --- | --- | --- | --- | --- | --- | --- | --- | --- | --- |
|  | **Tinn** | **Cont** | **I** | **III** | **V** | **I** | **III** | **V** | **I** | **III** | **V** | **I** | **III** | **V** |
| Santos-Filha et al. (2014) | 30 | 30 | 1.66 | 3.8 | 5.7 |  |  |  | 1.6 | 3.74 | 5.69 |  |  |  |
| Attias et al. (1996) | 13 | 11 | 1.33 | 3.50 | 5.37 | .09 | .25 | .35 | 1.35 | 3.61 | 5.47 | .14 | .24 | .37 |
| Attias et al. (1993) | 12 | 12 | 1.32 | 3.61 | 5.57 | .36 | .38 | .41 | 1.35 | 3.66 | 5.63 | .45 | .37 | .44 |
| Gilles et al. (2016) | 19 | 23 | 1.6 | 3.67 | 5.53 | .14 | .26 | .18 | 1.57 | 3.75 | 5.51 | .1 | .2 | .23 |
| Kim et al. (2016) | 123 |  | 1.64 | 3.83 | 5.84 |  |  |  |  |  |  |  |  |  |
| Nemati et al. (2014) | 25 | 16 |  |  |  | .28 | .35 | .58 |  |  |  | .32 | .36 | .5 |
| Cartocci et al. (2012) | 10 | 14 |  |  | 5.93 |  |  |  |  |  | 5.75 |  |  |  |
| Singh et al. (2011) | 25 | 20 | 1.59 | 3.62 | 5.43 |  |  |  | 1.53 | 3.68 | 5.54 |  |  |  |
| Maurizi et al. (1985) | 54 |  | 1.8 | 3.93 | 5.84 |  |  |  |  |  |  |  |  |  |
| Mahmoudian et al. 2013 | 44 | N/A | 1.95 | 3.98 | 6.02 |  | .13 | .29 | 1.90 | 3.99 | 5.97 |  | .13 | .32 |
| Gu et al. (2012) | 15 | 21 | 1.81 | 4.1 | 5.9 | .2 | .4 | .8 | 1.72 | 4.01 | 5.98 | .46 | .39 | .70 |
| Schaette et al. (2011) | 15 | 18 |  |  |  | .09 |  |  |  |  |  | .12 |  |  |
| Kehrle et al. (2008) | 37 | 38 | 1.58 | 3.76 | 5.59 |  |  |  | 1.46 | 3.65 | 5.41 |  |  |  |
| Rosenhall & Axelsson (1994) | 113 | 220 | 1.91 |  | 6.07 |  |  |  | 1.79 |  | 5.82 |  |  |  |
| Lemaire & Beutter (1995) | 355 | 129 | 1.77 | 3.97 | 5.87 | .15 | .20 | .33 | 1.62 | 3.77 | 5.6 | .27 | .33 | .42 |
| Barnea et al. (1990) | 12 | N/A | 1.43 | 3.63 | 5.54 | .32 | .37 | .37 |  |  |  |  |  |  |
| Ikner et al. (1990) | 35 | 35 | 1.88 | 3.93 | 5.91 |  |  |  | 1.76 | 3.8 | 5.72 |  |  |  |
| De Lavernhe-Lemaire & Beutter (1990) | 139 | 20 |  |  |  | .14 | .19 | .34 |  |  |  | .3 | .32 | .45 |
| De Lavernhe-Lemaire & Beutter (1989) | 164 | 57 | 1.77 | 3.97 | 5.85 |  |  |  | 1.59 | 3.77 | 5.59 |  |  |  |
